# A focused library synthesis and cytotoxicity of quinones derived from the natural product bolinaquinone

**DOI:** 10.1098/rsos.171189

**Published:** 2018-04-04

**Authors:** Azadeh Ghods, Jayne Gilbert, Jennifer R. Baker, Cecilia C. Russell, Jennette A. Sakoff, Adam McCluskey

**Affiliations:** 1 Chemistry, The University of Newcastle, University Drive Callaghan, Newcastle, New South Wales 2308, Australia; 2 Department of Medical Oncology, Calvary Mater Newcastle Hospital, Waratah, New South Wales 2298, Australia

**Keywords:** bolinaquinone, Suzuki coupling, microwave-assisted synthesis, cytotoxicity

## Abstract

Bolinaquinone is a natural product that is a structurally complex, cytotoxic sesquiterpene quinone. A scaffold simplification and focused library approach using a microwave-assisted Suzuki coupling gave 32 bolinaquinone analogues with good-to-excellent cytotoxicity profiles. Mono-arylbenzoquinones, Library **A**, were preferentially toxic towards BE2-C (neuroblastoma) cells with growth inhibition (GI_50_) values of 4–12 µM; only the 3,4-dimethoxyphenyl **23** and 3-biphenyl **28** variants were broad-spectrum active—HT29 (colon carcinoma), U87 and SJ-G2 (glioblastoma), MCF-7 (breast carcinoma), A2780 (ovarian carcinoma), H460 (lung carcinoma), A431 (skin carcinoma), Du145 (prostate carcinoma), BE2-C (neuroblastoma), MIA (pancreatic carcinoma) and SMA (spontaneous murine astrocytoma). Library **B** with a second aryl moiety exhibited broad-spectrum cytotoxicity with MCF-7 cells’ GI_50_ values of 5.6 ± 0.7 and 5.1 ± 0.5 µM for 2,5-dimethoxy-3-(naphthalene-1-yl)-6-(naphthalene-3-yl) **33** and 2,5-dimethoxy-3-(biaryl-2-yl)-6-(naphthalene-3-yl) **36,** respectively. Similar potencies were also noted with 2,5-dimethoxy-3,6-diphenyl **30** against A2780 (GI_50_ = 5.9 ± 0.0 µM) and with 2,5-dimethoxy-3-(biaryl-3-yl)-6-(naphthalene-3-yl) **37** against HT29 (GI_50_ = 5.4 ± 0.4 µM), while the 3,4-dimethoxy mono-aryl analogue **23** exhibited good levels of activity against A2780 (GI_50_ = 3.8 ± 0.75 µM), the neuroblastoma cell line BE2-C (GI_50_ = 3 ± 0.35 µM) and SMA (GI_50_ = 3.9 ± 0.54 µM). Introduction of the amino-substituted Library **C** gave 2-(naphthalen-1-yl)-5-(naphthalen-3-yl)-3,6-bis(propylamino) **43,** with excellent activity against HT29 (0.08 ± 0.0 µM), MCF-7 (0.17 ± 0.1 µM), A2780 (0.14 ± 0.1 µM), A431 (0.11 ± 0.0 µM), Du145 (0.16 ± 0.1 µM), BE2-C (0.08 ± 0.0 µM) and MIA (0.1 ± 0.0 µM).

## 1. Introduction

Cancer can be defined as a collection of diseases in which cells undergo aberrant division and at times invasive proliferation. Broadly speaking, there are over 100 cancer types spanning carcinoma, sarcoma, leukaemia, lymphoma and myeloma, and central nervous system cancers http://www.cancer.gov/cancertopics/cancerlibrary/what-is-cancer (accessed 25 May 2017). In Australia, cancer is the second most common cause of death after cardiovascular diseases with lifetime mortality risks of one in four for males and one in six for females http://www.cancer.org.au/about-cancer/what-is-cancer/facts-and-figures.html (accessed 25 May 2017). In 2014, there was an estimated 128 000 new Australian cancer diagnoses, a number predicted to reach 150 000 annually by 2020 [1].

Over the past decade, our group has explored two general approaches to the development of potential anti-cancer agents: (i) target-based approach and (ii) phenotypic screening that relies on cytotoxicity assays across a broad panel of cancer cell lines. Our target-derived explorations have led to the development of novel cytotoxic clathrin and dynamin GTPase [2–4], and protein phosphate 1 and 2A inhibitors [5–8]. Our phenotypic approach has identified the cytotoxic acrylonitriles [9,10], steroids [11], natural product-derived leads [12,13] and more recently inhibitors of the hedgehog signalling pathway [14,15].

**Figure 1.**
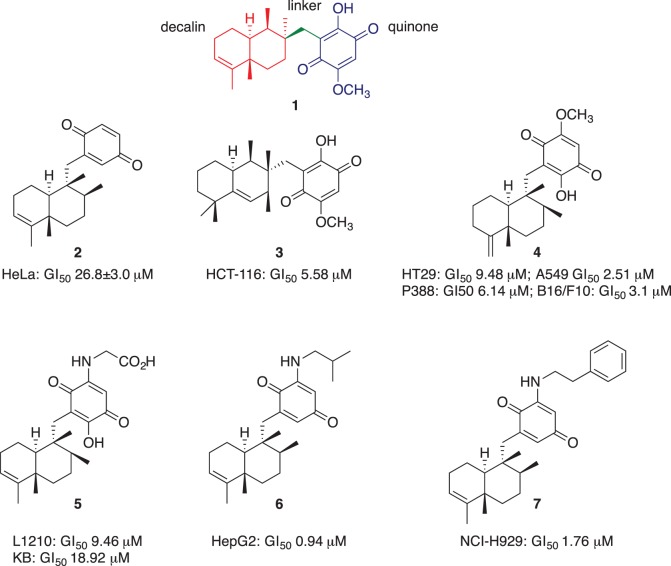
The chemical structures of selected sesquiterpene quinones: bolinaquinone (**1**), avarone (**2**), mamanuthaquinone (**3**), epi-ilimaquinone (**4**), nakijiquinone A (**5**), dysidaminone E-5 (**6**) and 18-phenethylaminoavarone (**7**), and their reported cytotoxicity towards different cancer cell lines.

Given that the phenotypic screening has seen resurgence in use over the past few years [16], we sought to expand our efforts in this area. Here our interest focused on the cytotoxic natural product bolinaquinone (**1**, figure 1). Bolinaquinone has been shown to inhibit the growth of HCT-116 colon carcinoma cells with a GI_50_ = 5.3 µM (50% growth inhibition) [17]. Cytotoxicity has also been reported for related marine sesquiterpene quinones such as avarone (**2**), mamanuthaquinone (**3**), epi-ilimaquinone (**4**), nakijiquinone A (**5**), dysidaminone E-5 (**6**) and 18-phenethylaminoavarone (**7**) (figure 1). These quinones also reveal a wide range of cytotoxicity against a diverse panel of cancer cell lines: **2**: HeLa (GI_50_ = 26.8 ± 3.0 µM), L5178Y mouse lymphoma cells (ED_50_ = 0.62 ± 0.11 µM) [18,19]; **3**: HCT-116 (GI_50_ = 5.58 µM) [20]; **4**: P-388 leukaemia cells (GI_50_ = 6.14 µM) and different solid tumours: A-549 lung (GI_50_ = 2.51 µM), HT-29 colorectal (GI_50_ = 9.48 µM) and melanoma B16/F10 (GI_50_ = 3.1 µM) [21]; **5**: leukaemia L-1210 and epidermoid KB cell lines with (GI_50_ = 9.46 and 18.92 µM, respectively) [22]; **6**: HepG2 hepatoma cancer cell line (GI_50_ = 0.94 µM) and **7**: NCI-H929 myeloma (GI_50_ = 1.76 µM) [23]. Quinones have been previously explored in clinical development [24,25] and are believed to elicit their cytotoxic action via a redox cycling mechanism. Alongside this mode of action, quinones are reported to be promiscuous, especially those analogues capable of both redox cycling (electron scavenging) and forming Michael adducts [26,27]. Despite the potential PAIN nature of quinones, it has been proposed that they be retained in phenotypic screening libraries, with a key argument being that the core motif is present in numerous clinically used drugs, e.g. the anthracyclines, mitomycin and mitoxantrone. Despite their mechanism of action remaining unknown, these agents are successfully used in the treatment of multiple cancers, pneumonia and multiple sclerosis. Quinone potentially provide leads, but these require careful examination for promiscuity, which arguably is the role of medicinal chemistry—to remove toxicity and enhance selectivity [28].

Synthetic routes to sesquiterpenes (**1**–**7**) are either complex and low yielding, or yet to be reported [29–32]. Thus, herein we explore a scaffold simplification approach in our efforts to develop the cytotoxicity structure–activity relationship of bolinaquinone analogues.

## 2. Procedures

### 2.1. Synthetic

**Figure 2.**
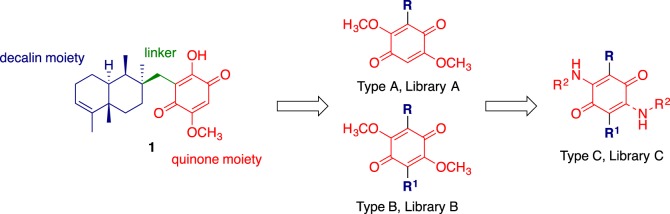
The proposed structural simplification of the lead, bolinaquinone **1**, to afford three bolinaquinone libraries: **A**, **B** and **C**.

We viewed our lead, bolinaquinone (**1**) as comprising three readily modifiable regions: the decalin or hydrophobic moiety, the linker and quinone moieties (figure 2). Based on this, three focused libraries were designed to explore the effect of the modification of each region. In our initial design considerations, given that there was no prior structure activity data in this area, we were keen to simplify access to bolinaquinone analogues. To this end, we envisaged analogues that retained the central quinone core with at least one methoxy moiety and a range of pendant aromatic moieties fulfilling the role of the hydrophobic decalin moiety of **1**. The lack of a polar moiety associated with the pendant parent decalin of **1** and analogues **2**–**7** supported the introduction of simple aromatic moieties only. We were cognisant of the potentially detrimental effect on compound physico-chemical properties on the introduction of multiple lipophilic groups, but viewed that this may also improve cellular uptake. Thus Library **A** sought to explore the effect of decalin core replacement with less complex hydrophobic moieties such as simple aromatic rings (Type **A** analogues, figure 2). This route allowed the use of well-established Suzuki coupling approaches [33,34]. The introduction of a methoxy moiety offered the possibility of synthesis simplification and direct access to Library **B** through further scaffold arylation (Type **B** analogues, figure 2); in doing so, the hydroxyl moiety of **1** was modified to a methoxy moiety (Type **A** analogues, figure 2). Finally, in the development of dynamin inhibitors we had shown that the introduction of amino moieties increased inhibition and analogue solubility, and viewed that this approach would potentially enhance cytotoxicity and ameliorate the poor physico-chemical characteristics of the hydrophobic analogues planned in Libraries **A** and **B**. Thus amine displacement of the methoxy moieties afforded Library **C** (Type **C** analogues, figure 2).

**Scheme 1.**
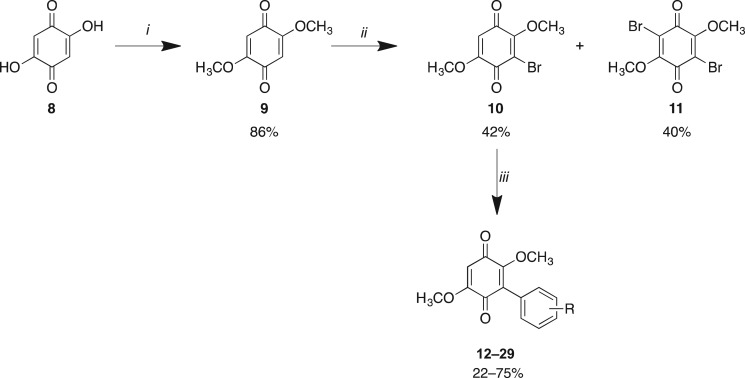
Reagents and conditions: (*i*) MeOH, H_2_SO_4_, 25°C, 6 h; (*ii*) NBS, DMF, 60°C (then 25°C), 5–8 h; (*iii*) arylboronic acid (Ar-B(OH)_2_), Pd(dppf)Cl_2_, K_2_CO_3_, dioxane, µW, 120°C, 20 min, or arylboronic acid (R-B(OH)_2_), Pd(dppf)Cl_2_, K_2_CO_3_, toluene, reflux, 72 h.

Library **A** synthesis commenced with the treatment of 2,5-dihydroxy-1,4-benzoquinone (**8**) in methanol in the presence of catalytic H_2_SO_4_ at room temperature. This afforded, after work-up, the desired 2,5-dimethoxy-1,4-benzoquinone (**9**) in an 86% yield [35]. Bromination of **9** through warming the reaction mixture in DMF at 60°C (then 25°C) with portion-wise addition of excess of *N*-bromosuccinimide (NBS) gave a readily separable mixture of 3-bromo-2,5-dimethoxy-1,4-benzoquinone (**10**) and 3,6-dibromo-2,5-dimethoxy-1,4-benzoquinone (**11**) in a 42% and 40% yield, respectively (scheme 1) [36,37].

By varying the bromination conditions, preferential access to the mono- or dibrominated products was possible. This simplified product isolation. For 3-bromo-2,5-dimethoxy-1,4-benzoquinone (**10**), the quinone was dissolved in DMF and heated to 100°C followed by the addition of 1 equivalent of NBS in a single portion. The dibrominated product, 3,6-dibromo-2,5-dimethoxy-1,4-benzoquinone (**11**), was accessed through the addition of 2 equivalents of NBS in DMF at 60°C. In both approaches, the desired product was isolated by flash chromatography. Subsequent application of a microwave-assisted Suzuki coupling approach with **10** gave facile access to Library **A** analogues **12**–**29** [33]. With the exception of sterically encumbered and aliphatic boronic acids, e.g. anthracene and cyclohexyl, microwave irradiation at 120°C for 20 min gave these analogues in 60–75% yield (scheme 1 and table 1). Within this library those reactions that displayed poor conversion to the desired analogue (**14**, **18**, **19**, **22** and **23**) were also accessed by reflux approaches as extended microwave irradiation led to increasing levels of the undesired des-methoxy analogues. In general, these batch reactions were lower yielding when compared with the microwave conditions. Moreover, we noted that (in our hands) the Suzuki coupling failed to provide useful quantities of the target aryl-coupled quinones with the 2-hydroxy-, 3-hydroxy- and 4-methoxy- phenyl boronic acids. The 2-tolyl boronic acid analogue failed to react to completion and gave rise to a complex, inseparable mixture; as such, this analogue was not pursued further.

**Table 1.** Evaluation of the cytotoxicity, GI_50_ values (µM) of 3-aryldimethoxybenzoquinone derivatives **12**–**29** (Library **A**), against a panel of 11 cancer cell lines and one normal cell line. GI_50_ is the concentration of drug that reduces cell growth by 50% relative to an untreated control.

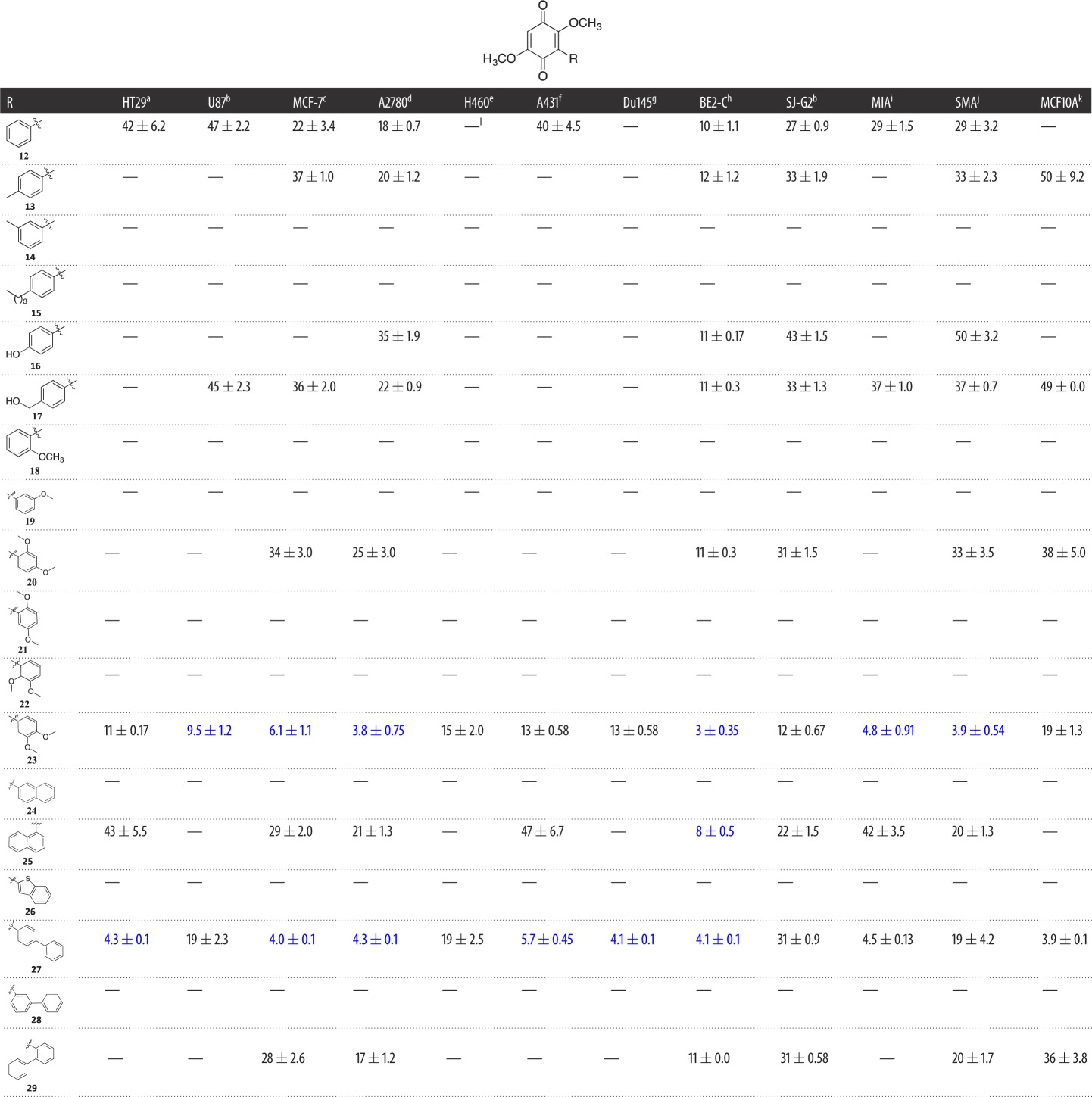

^a^HT29 (colon carcinoma).

^b^U87 and SJ-G2 (glioblastoma).

^c^MCF-7 (breast carcinoma).

^d^A2780 (ovarian carcinoma).

^e^H460 (lung carcinoma).

^f^A431 (skin carcinoma).

^g^Du145 (prostate carcinoma).

^h^BE2-C (neuroblastoma).

^i^MIA (pancreatic carcinoma).

^j^SMA (spontaneous murine astrocytoma).

^k^MCF10A (normal breast cell line).

^l^‘—’ = compounds screened only at a single dose, 25 µM and not sufficiently active to proceed to full dose response—these data are provided in the electronic supplementary material.

**Scheme 2.**
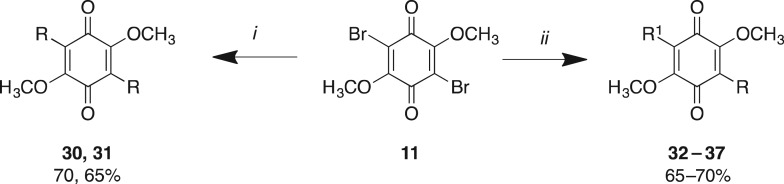
Reagents and conditions: (*i*) 2 equivalents of arylboronic acid (R-B(OH)_2_), Pd(dppf)Cl_2_, K_2_CO_3_, dioxane, μW, 120°C, 20 min; (*ii*) 1 equivalent of arylboronic acid (R-B(OH)_2_), Pd(dppf)Cl_2_, K_2_CO_3_, dioxane, μW, 120°C, 20 min, then 1 equivalent of arylboronic acid (R'-B(OH)_2_), 120°C, 20 min.

Library **B** leveraged our ability to simultaneously conduct a Suzuki coupling with either two equivalents of an arylboronic acid or sequentially with one equivalent of two different arylboronic acids (scheme 2). Symmetrical 3,6-diaryl-dimethoxybenzoquinones were synthesized on treatment of **11** with two equivalents of an arylboronic acid and microwave irradiation at 120°C for 20 min as previously described, which afforded **30** and **31**. Asymmetric analogues **32–37** were accessed by treatment of **11** with one equivalent of arylboronic acid under microwave conditions, and once TLC analysis had confirmed consumption of the starting materials, with ensuing addition of an equivalent of a second arylboronic acid and microwave irradiation at 120°C for 20 min as previously described. Analogues **30–37** were obtained in 65–70% yields after flash chromatography (scheme 2 and table 2). In some instances these Suzuki couplings failed, e.g. with the 1-naphthyl, 2-naphthyl and 4-biphenyl boronic acids as one of the substituents, which was most probably a consequence of steric hindrance. The asymmetric analogues were purified only in the last step to omit time-consuming purification of intermediates.

**Table 2.** Evaluation of the cytotoxicity, GI_50_ values (μM) of 3-aryldimethoxybenzoquinone derivatives **30**–**37** (Library **B**), against a panel of 11 cancer cell lines and one normal cell line. GI_50_ is the concentration of drug that reduces cell growth by 50% relative to an untreated control.

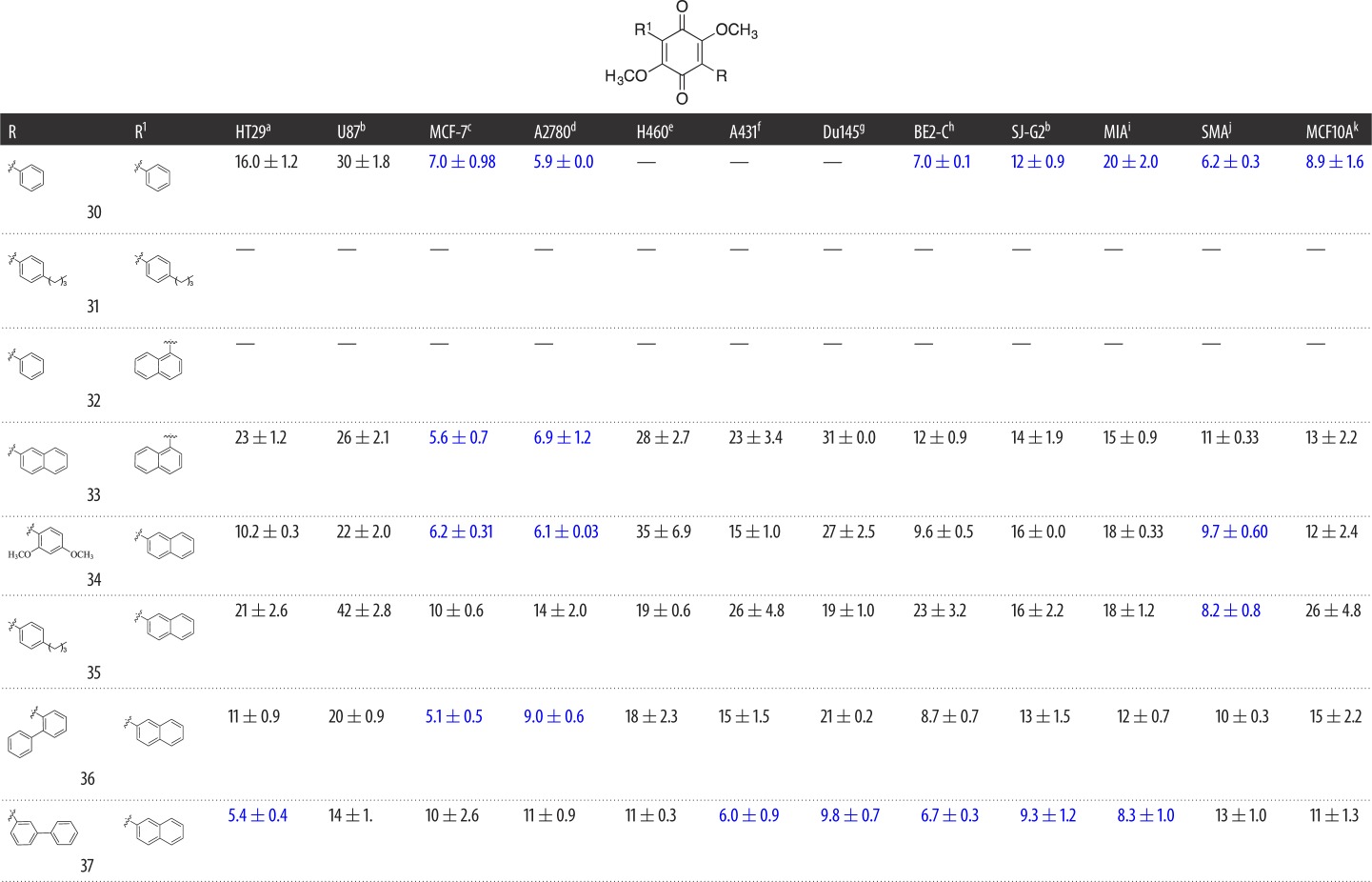

^a^HT29 (colon carcinoma).

^b^U87 and SJ-G2 (glioblastoma).

^c^MCF-7 (breast carcinoma).

^d^A2780 (ovarian carcinoma).

^e^H460 (lung carcinoma).

^f^A431 (skin carcinoma).

^g^Du145 (prostate carcinoma).

^h^BE2-C (neuroblastoma).

^i^MIA (pancreatic carcinoma).

^j^SMA (spontaneous murine astrocytoma).

^k^MCF10A (normal breast cell line).

**Scheme 3.**
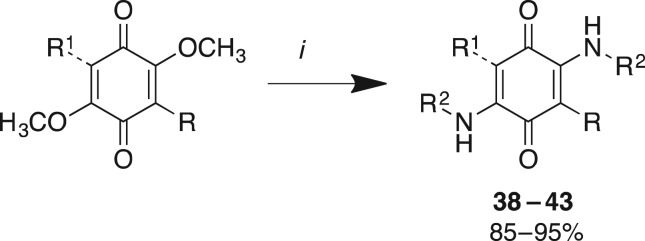
Reagent and conditions: (*i*) *n*-propyl amine or *N,N*-dimethylpropane-1,3-diamine, MeOH, 25°C, 1 h.

In the construction of Library **C**, a total of six analogues from Library **A** and Library **B** were treated with either *n*-propyl amine or *N,N*-dimethylpropane-1,3-diamine in methanol at room temperature to afford excellent yields (85%–95%) of the diaminoquinones (**38–43**) (scheme 3 and table 3).

**Table 3.** Evaluation of the cytotoxicity, GI_50_ values (μM) of 3-aryldimethoxybenzoquinone derivatives **38**–**43** (Library **C**), against a panel of 11 cancer cell lines and one normal cell line. GI_50_ is the concentration of drug that reduces cell growth by 50% relative to an untreated control.

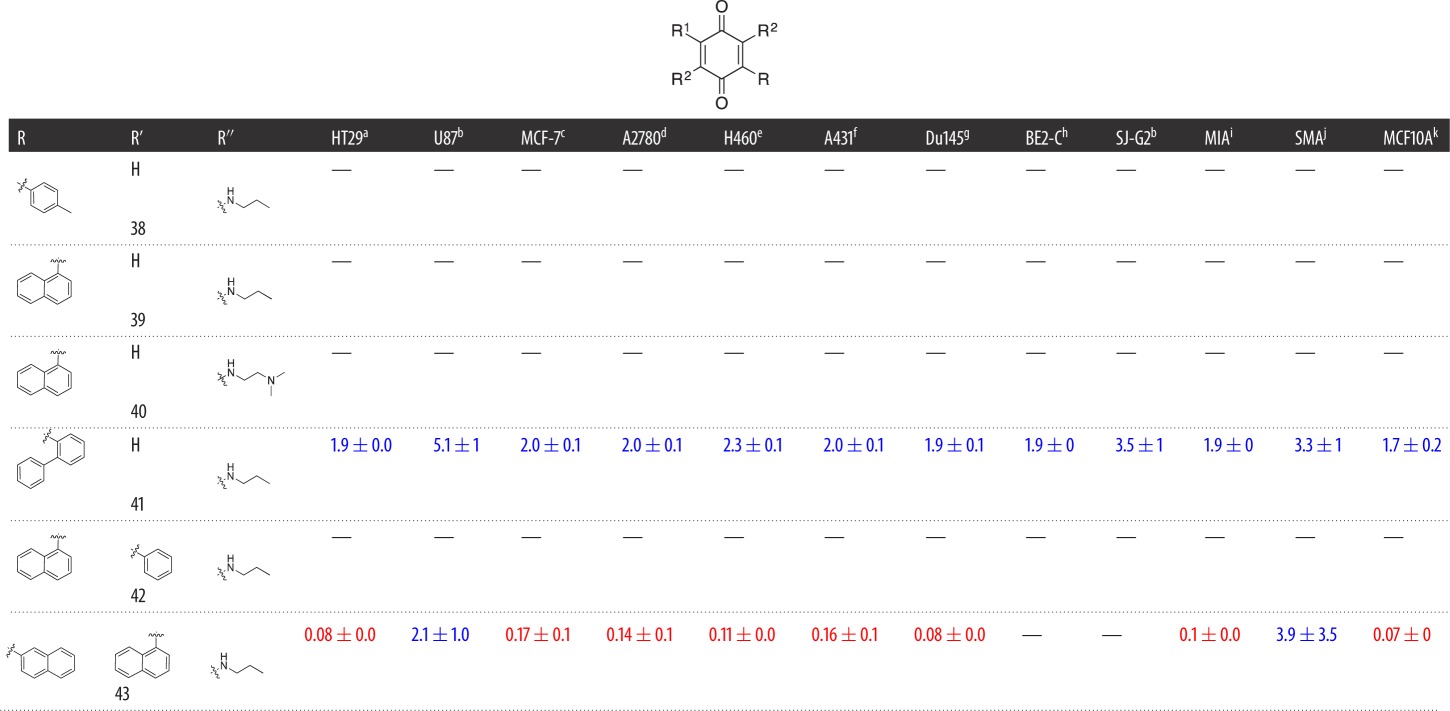

^a^HT29 (colon carcinoma).

^b^U87 and SJ-G2 (glioblastoma).

^c^MCF-7 (breast carcinoma).

^d^A2780 (ovarian carcinoma).

^e^H460 (lung carcinoma).

^f^A431 (skin carcinoma).

^g^ Du145 (prostate carcinoma).

^h^BE2-C (neuroblastoma).

^i^MIA (pancreatic carcinoma).

^j^SMA (spontaneous murine astrocytoma).

^k^MCF10A (normal breast cell line).

### 2.2. Cytotoxicity

With the desired bolinaquinone analogues **12–43** in hand, we examined their cytotoxicity against a panel of 11 tumour cell lines: HT29 (colon carcinoma), U87 and SJ-G2 (glioblastoma), MCF-7 (breast carcinoma), A2780 (ovarian carcinoma), H460 (lung carcinoma), A431 (skin carcinoma), Du145 (prostate carcinoma), BE2-C (neuroblastoma), MIA (pancreatic carcinoma), SMA (spontaneous murine astrocytoma) and a normal breast cell line (MCF10A). The inclusion of the MCF-10A cell line allowed a determination of a crude measure of relative toxicity while noting that the ultimate arbiter of toxicity requires *in vivo* evaluation [38,39]. All analogues were initially screened at a 25 µM drug concentration [40,41]. Those analogues returning 80–100% growth inhibition across all cell lines, or those displaying cell line specificity were subjected to full dose response, GI_50_, evaluation. These data are given in tables 1–3.

## 3. Results and discussion

Our investigation of the bolinaquinone SAR commenced with an application of a scaffold simplification and focused library approach that saw the synthesis of mono-arylated dimethoxybenzoquinones (Library **A**). Of this initial library, analogues **14**, **15**, **18**, **19**, **21**, **22**, **24, 26** and **28** were deemed not sufficiently active at the initial screening concentration of 25 µM to proceed to GI_50_ determination (table 1; electronic supplementary material). Of the other Library **A** members, phenyl **12** and 4-toluoyl **13** were essentially equipotent across the cell line panel with GI_50_ values ranging from 10 ± 1.1 µM, (**12**, BE2-C) to greater than 50 µM (**12**, H460; and **13**, HT29, U87, H460, A431 and MIA). However, **13** showed no activity (defined here as a GI_50_ value greater than 50 µM) against HT29, U87, A431 and MIA cell lines, and was 50 µM potent against the normal cell line, MCF10A. The 3-toluoyl **14** analogue was inactive as was 4-butyl **15,** which suggested that the position and nature of the simple aliphatic substituent affected the potency of these analogues with a 3-alkyl moiety and a larger 4-alkyl moiety not tolerated. Polar substituents on the introduced phenyl moiety such as 4-OH **16**, 4-CH_2_OH **17** and 2,4-di-OCH_3_ **20** showed moderate activity against BE2-C with all GI_50_ values approximately 11 µM, and were equipotent with **12** and **13** with similar activity profiles across the cell lines examined. The mono-OCH_3_ analogues **18** and **19** were inactive. Of the di-OCH_3_ analogues **20**–**23** examined, only 3,4-di-OCH_3_ **20** was active. Notably, of analogues **20**–**23**, analogue **20** was the only active 2-substituted di-OCH_3_ analogue, albeit modestly (except against BE2-C cells), which suggests that a 2-substituent may be detrimental to activity. The most active of the compounds with single aromatic moieties on the quinone scaffold was found to be 3,4-di-OMe **23**, which was also the first broad-spectrum cytotoxic analogue in this series displaying GI_50_ values of 3–15 µM across the cancer cell lines examined.

Within Library **A** the introduction of a larger aromatic moiety resulted in a modest increase in cytotoxicity with 1-naphthyl **25** showing preferential activity towards the BE2-C cell line, with a GI_50_ value of 8 ± 0.5 µM; however, 2-naphthyl **24** was inactive. In a similar manner, the benzothiophene **26** and 3-biphenyl **28** analogues did not proceed to GI_50_ determination. However, the introduction of a 2- and especially a 4-biphenyl moiety with **29** and **27**, respectively, resulted in increased cytotoxicity with the latter returning broad-spectrum activity from 3.9 to 19 µM (table 1), but also a significant increase in toxicity towards the MCF10A cell line with **27** returning a GI_50_ of 3.9 µM. This in turn suggested that the orientation of the aromatic or hydrophobic moiety relative to the core quinone moiety is critical to the retention of cytotoxicity. The two most active compounds in this library, 4-biphenyl **27** and 3,4-di-OMe **23,** both contain 4-disposed moieties. The introduction of a 2-substituent results in the aromatic moieties twisting out of plane relative to the quinone moiety, e.g. **18** and **20**–**22**, which has an adverse impact on the observed cytotoxicity (not shown).

Library **A** demonstrated that the introduction of large aromatic moieties resulted in good levels of broad-spectrum cytotoxicity with **27**; as such, with Library **B** we sought to explore the effect of increasing the aromatic content of these analogues on compound cytotoxicity. Interestingly, the introduction of a second aryl moiety with Library **B** gave rise to a different activity profile to that observed with Library **A**. The parent diphenyl (**30**) analogue showed moderate-to-good broad-spectrum cytotoxicity, e.g. A2780 GI_50_ = 5.9 ± 0.0 µM and SMA GI_50_ = 6.2 ± 0.3 µM, except with the H460, A431 and Du145 cell lines (table 2; GI_50_ > 50 µM). The introduction of an alkyl chain with **31** was detrimental to activity (table 2). However, this did not appear to be a consequence of a steric clash as the naphthyl substituted **32–37**, even in the presence of the 2- and 3-biphenyl moieties, returned good-to-excellent cytotoxicity across the cell lines examined. In these instances, the GI_50_ values ranged from 5.1 ± 0.5 µM (**36**; MCF-7) to 35 ± 6.9 µM (**34**; H460). The bis-naphthyl (**33**) saw an overall reduction in cytotoxicity, except with the ovarian and breast cell lines MCF-7 and A2780, with GI_50_ values of 5.6 ± 0.7 and 6.9 ± 1.2 µM, respectively. The replacement of the decalin moiety of **1** with a phenyl moiety (**12**) resulted in a decrease in cLogP (5.10 to 1.85) and polar surface area (PSA, 48.97 to 41.65 Å^2^); a 4-biphenyl (**27**) moiety resulted in cLogP of 3.65 and a PSA of 41.90 Å^2^ (see the electronic supplementary material). The introduction of a second aromatic moiety was highly detrimental to cLogP with 37 displaying a cLogP of 6.71. However, as we suspected from our earlier dynamin studies, the introduction of amino moieties improved the cLogP values of the resultant analogues such as **41** (cLogP 5.21), but the presence of the amino moieties failed to overcome the effect of two naphthyl moieties with **43** (cLogP 7.91). Despite this significant deterioration in cLogP, we noted no effect of compound precipitation during our cytotoxicity studies, with all analogues soluble in DMSO at 40 mM and maintaining solubility in the MTT assay.

Having established the effects of aryl substitutions on the cytotoxicity of modified bolinaquinone analogues, we next examined the displacement of the –OCH_3_ moieties with simple amines to generate Library **C**. Of the six Library **C** analogues, only **41** and **43** returned sufficient activity at the initial 25 µM screening dose to proceed to GI_50_ determination (table 3). However, both **41** and **43** displayed considerable increase in cytotoxicity, returning the highest level of activity of any of the analogues developed thus far with average GI_50_ values of 2.5 µM and 0.69 µM, respectively. Comparison of **41** and **43** with the parent analogues **29** (table 1) and **33** (table 2), respectively, highlights a greater than or equal to 20-fold potency enhancement on the introduction of the amine substituents, potentially suggesting the presence of additional binding domains that these substituents are capable of accessing. Of **41** and **43**, the tri-substituted quinone moiety of **41** is capable of both DNA adduction and redox cycling, whereas the tetra-substituted quinone moiety of **43** is not.

While the exact cytotoxic mechanism was not determined, these data support **43** mediating its effects through a redox-cycling mechanism and validate our phenotypic approach to the identification of cytotoxic compounds. Within the cell lines examined, **41** displayed no obvious cell line preference. The bis-naphthylamino-substituted **43** analogue returned nanomolar potency against HT29 (colon), MCF-7 (breast), A2780 (ovarian), H460 (lung), A431 (skin), Du145 (prostate) and MIA (pancreatic). However, while **43** returned a GI_50_ value of 0.07 ± 0 µM against the normal breast cell line MCF-10A, it is critical to note that an *in vitro* assay is a poor determinant of human toxicity, noting that compound toxicity can only really be measured *in vivo* [38]. It is accepted that animal models are required to predict potential clinical toxicity.

## 4. Conclusion

The natural product bolinaquinone was reported as moderately cytotoxic (HCT-116, GI_50_ = 5.3 µM). The lead was deemed to be structurally too complex to facilitate rapid focused library development while retaining the parent hydrophobic core. However, application of a scaffold simplification approach focusing on the hydrophobic core combined with a microwave-assisted Suzuki coupling (and, in some cases, reflux conditions) permitted the synthesis of 32 modified bolinaquinone analogues. These novel analogues spanned three focused libraries. Simple mono-arylbenzoquinones displayed preferential toxicity towards the BE2-C neuroblastoma cell line with GI_50_ values of 3–12 µM, apart from the 3,4-dimethoxy **(23)** and 4-biphenyl (**27**), which returned broad-spectrum cytotoxicity with an average GI_50_ of 10.2 µM. The introduction of a second aryl moiety with Library **B** failed to enhance BE2-C specificity, but resulted in enhanced broad-spectrum cytotoxic activity in general. Despite the transition towards broad-spectrum cytotoxicity, good levels of activity were apparent against the MCF-7 breast cancer cell lines with GI_50_ values of 5.6 ± 0.7 and 5.1 ± 0.5 for **33** and **36,** respectively. Similar potencies were also noted with **30** against the ovarian cell line A2780 (GI_50_ = 5.9 ± 0.0 µM), with **37** against the colon cell line HT29 (GI_50_ = 5.4 ± 0.4 µM), and with **23** against the ovarian cell line A2780 (GI_50_ = 3.8 ± 0.75 µM), the neuroblastoma cell line BE2-C (GI_50_ = 3 ± 0.35 µM) and the glioblastoma cell line SMA (GI_50_ = 3.9 ± 0.54 µM).

Introduction of the amino substituents with Library **C** resulted in the development of the most potent broad-spectrum analogues with **41** and **43** yielding average GI_50_ values of 2.3 and 0.69 µM, respectively. As previously noted herein, enhanced broad-spectrum activity resulted from the introduction of a second aryl moiety, as is the case with **41** and **43**. In addition, **43** displayed excellent activity against HT29 (0.08 ± 0.0 µM), MCF-7 (0.17 ± 0.1 µM), A2780 (0.14 ± 0.1 µM), A431 (0.11 ± 0.0 µM), Du145 (0.16 ± 0.1 µM), BE2-C (0.08 ± 0.0 µM) and MIA (0.1 ± 0.0 µM). Of the analogues reported herein, the highest levels of cytotoxicity were observed in those with bulky aromatic moieties such as a biphenyl and naphthyl, especially in the presence of additional propyl amine moieties. These modified bolinaquinone analogues are promising leads in the search for new cytotoxic agents and we will report on further developments in due course.

## 5. Experimental

### 5.1. Biology

#### 5.1.1. Cell culture and stock solutions

Stock solutions were prepared as follows and stored at −20°C: drugs were stored as 40 mM solutions in DMSO. All cell lines were cultured in a humidified atmosphere 5% CO_2_ at 37°C. The cancer cell lines were maintained in Dulbecco's modified Eagle's medium (DMEM) (Trace Biosciences, Australia) supplemented with 10% fetal bovine serum, 10 mM sodium bicarbonate, penicillin (100 IU ml^−1^), streptomycin (100 µg ml^−1^) and glutamine (4 mM). The non-cancer MCF10A cell line was cultured in DMEM:F12 (1 : 1) cell culture media, 5% heat-inactivated horse serum, supplemented with penicillin (50 IU ml^−1^), streptomycin (50 µg ml^−1^), 20 mM Hepes, L-glutamine (2 mM), epidermal growth factor (20 ng ml^−1^), hydrocortisone (500 ng ml^−1^), cholera toxin (100 ng ml^−1^) and insulin (10 ug ml^−1^).

#### 5.1.2. *In vitro* growth inhibition assay

Cells in logarithmic growth were transferred to 96-well plates. Cytotoxicity was determined by plating cells in duplicate in 100 µl medium at a density of 2500–4000 cells per well. On day 0 (24 h after plating), when the cells were in logarithmic growth, 100 μl of medium with or without the test agent was added to each well. After 72 h, drug exposure growth inhibitory effects were evaluated using the MTT (3-[4,5-dimethylthiazol-2-yl]-2,5-diphenyl-tetrazolium bromide) assay and the absorbance read at 540 nm. Percentage growth inhibition was determined at a fixed drug concentration of 25 µM. A value of 100% is indicative of complete cell growth inhibition. Those analogues showing appreciable percentage growth inhibition underwent further dose–response analysis allowing for the calculation of a GI_50_ value. This value is the drug concentration at which cell growth is 50% inhibited based on the difference between the optical density values on day 0 and those at the end of drug exposure [40,41].

### 5.2. Chemistry

#### 5.2.1. General methods

All reactions were performed using standard laboratory equipment and glassware. Solvents and reagents were purchased from Sigma Aldrich, Lancaster International or TCI and used as received. Organic solvents were of bulk quality, and were distilled from glass prior to use. Organic solvent extracts were dried with magnesium sulfate (MgSO_4_), and dried under reduced pressure with either Büchi or Heidolph rotary evaporators. Melting points were recorded in open capillaries on a Stuart SMP11 melting point apparatus. Where available, literature values are provided and appropriately referenced. Electrospray mass spectra were recorded using 10% DMSO/H_2_O or HPLC-grade methanol or acetonitrile as carrier solvents on a Shimadzu LC-MS spectrometer, or an Agilent Technologies 1260 Infinity UPLC system with a 6120 Quadrupole LC/MS in electrospray ionization (ESI) positive and negative modes. TLC was performed on Merck silica gel 60 F_254_ pre-coated aluminium plates with a thickness of 0.2 mm. Column chromatography was performed under ‘flash’ conditions on Merck silica gel 60 (230–400 mesh).

Nuclear magnetic resonance (NMR) spectroscopy was performed on a Brüker Avance III 400 MHz spectrometer, where proton NMR (^1^H NMR) spectra and carbon NMR (^13^C NMR) spectra were acquired at 400 and 100 MHz, respectively. All spectra were recorded in deuterated dimethyl sulfoxide (DMSO-*d_6_*), deuterated acetone (acetone-*d_6_*) or deuterated chloroform (CDCl_3_) obtained from Sigma Aldrich or Cambridge Isotope Laboratories Inc. Chemical shifts (*δ*) were measured in parts per million (ppm) and referenced against the internal reference peaks. Coupling constants (*J*) were measured in hertz (Hz). NMR assignments were determined through the interpretation of one- and two-dimensional spectra. Multiplicities are denoted as singlet (s), broad singlet (s, br), doublet (d), doublet of doublets (dd), triplet (t), quartet (q), triplet of doublets (td), doublet of triplets (dt) and multiplet (m). Peaks are listed in decreasing chemical shift in the following format: chemical shift (integration (^1^H), multiplicity (^1^H), coupling constant (^1^H). The Biotage^®^ initiator^+^ was used to perform Suzuki coupling (Technical information available on: http://www.biotage.com).

Synthesis of 2,5-dimethoxycyclohexa-2,5-diene-1,4-dione (**9**).

To a solution of 2,5-dihydroxy-1,4-benzoquinone **8** (2.80 g, 20.0 mmol) in MeOH (150 ml), H_2_SO_4_ (98%, 4 ml) was added dropwise, and the resultant mixture allowed to stir at room temperature for 6 h. The precipitate was collected, washed with MeOH : water (9 : 1, 2 × 20 ml) and dried *in vacuo*. The title compound was obtained as a yellow solid with identical spectral data to the previous report [29]. Yield 2.89 g (86%); m.p. 245–247°C (Lit. 240–242°C) [31].

^1^H NMR (DMSO-*d_6_*) *δ* 6.03 (s, 2H), 3.78 (s, 6H); ^13^C NMR (DMSO-*d_6_*) *δ* 181.9 (2C), 159.7 (2C), 106.0 (2C), 57.5 (2C); IR *υ*_max_/cm^−1^: 1652 (C=O), 1579 (C=C), 1328 (C–O); LRMS (ESI^+^) *m/z*: 168 (M^+^).

Synthesis of 3-bromo-2,5-dimethoxycyclohexa-2,5-diene-1,4-dione (**10**) and 3,6-dibromo-2,5-dimethoxycyclohexa-2,5-diene-1,4-dione (**11**).

A suspension of **9** (0.34 g, 2.02 mmol) in DMF (15 ml) was stirred for 10 min at 60°C and then NBS (0.79 g, 4.50 mmol) was added quickly in one portion to the solution. The reaction mixture was cooled to room temperature (25°C) and stirred for 8 h. Next, water (50 ml) was added and the mixture was extracted with EtOAc (2 × 30 ml), dried over MgSO_4_ and the solvent evaporated *in vacuo*. The crude product was purified by flash chromatography (5% EtOAc—95% hexanes). The title compounds were obtained as orange and red crystalline solids, respectively, with identical spectral data to the previous report [31,32].

(**10**) Yield 0.21 g (42%); m.p. 135–137°C (Lit. 136–138°C) [31]. ^1^H NMR (DMSO-*d_6_*) *δ* 5.99 (s, 1H), 4.21 (s, 3H), 3.89 (s, 3H); ^13^C NMR (DMSO-*d_6_*) *δ* 180.8, 175.0, 158.8, 157.5, 114.5, 105.5, 61.3, 56.6; IR *υ*_max_/cm^−1^: 1647 (C=O), 1557 (C=C), 1331 (C–O); LRMS (ESI^+^) *m/z*: 247 (M^+^).

(**11**) Yield 0.26 g (40%); m.p. 166–168°C (Lit. 168–170°C) [31]. ^1^H NMR (DMSO-*d_6_*) *δ* 4.10 (s, 6H); ^13^C NMR (DMSO-*d_6_*) *δ* 174.9 (2C), 157.1 (2C), 115.4 (2C), 62.2 (2C); IR *υ*_max_/cm^−1^: 1651 (C=O), 1558 (C=C), 1330 (C–O); LRMS (ESI^+^) *m/z*: 325 (M^+^).

General procedure for synthesis of 2,5-dimethoxy-3-aryl-2,5-diene-1,4-diones by Suzuki coupling (**1**).

(**A**) A Biotage microwave vial was charged with **10** (0.25 g, 1.01 mmol), requisite boronic acid (1.20 mmol), K_2_CO_3_ (0.34 g, 2.50 mmol) and Pd(dppf)Cl_2_ (0.07 g, 0.01 mmol), and then dioxane (5 ml) was added to this mixture. The reaction vessel was sealed and microwave-irradiated at 120°C for 20 min (holding time) with magnetic bar stirrer. After cooling with compressed air to 40°C, the reaction mixture was diluted with dioxane (10 ml) and filtered through the Celite®. The reaction crude was dried *in vacuo* and subjected to column chromatography for purification (15% EtOAc–85% hexanes) to afford an orange-red solid.

(**B**) Where none of the desired product or large amounts of starting material was found after microwave irradiation, the reactions were investigated under reflux conditions. A mixture of **10**, the requisite boronic acid (1.20 equiv), K_2_CO_3_ (2.5 equiv), Pd(dppf)Cl_2_ (0.2 equiv) and toluene (15 ml) was heated at reflux and monitored by TLC and LCMS until the starting material had been consumed. When the reaction was deemed complete, the reaction mixture was absorbed onto silica and purified by automated column chromatography (0–100% EtOAc in hexanes).

2,5-Dimethoxy-3-phenylcyclohexa-2,5-diene-1,4-dione (**12**).

Synthesized according to the general procedure 1A from phenyl boronic acid (0.14 g, 1.20 mmol). The title compound was obtained as a yellow solid with identical spectral data to the previous report [30]. Yield 0.18 g (73%); m.p. 122–124°C (Lit. 124–126°C) [31].

^1^H NMR (acetone-*d*_6_) *δ* 7.43–7.36 (m, 3), 7.31–7.28 (m, 2H), 6.01 (s, 1H), 3.88 (s, 3H), 3.86 (s, 3H); ^13^C NMR (acetone-*d*_6_) *δ* 183.2, 181.2, 158.9, 155.3, 131.0, 130.5, 128.5, 128.0, 127.5, 125.9, 124.6, 105.7, 60.8, 56.1; IR *υ*_max_/cm^−1^: 1648 (C=O), 1587 (C=C), 1332 (C–O); LRMS (ESI^+^) *m/z*: 245 (M+H), HRMS (ESI^+^) *m/z* calculated for C_14_H_13_O_4_ (M+H) 245.0813; found 245.0810.

2,5-Dimethoxy-3-p-tolylcyclohexa-2,5-diene-1,4-dione (**13**).

Synthesized according to the general procedure 1A from p-tolylboronic acid (0.16 g, 1.20 mmol). The title compound was obtained as an orange solid. Yield 0.19 g (74%); m.p. 121–123°C.

^1^H NMR (acetone-*d_6_*) *δ* 7.23 (d, J = 7.7 Hz, 2H), 7.18 (dd, *J* = 7.72, 2.2 Hz, 2H), 6.00 (s, 1H), 3.88 (s, 3H), 3.85 (s, 3H), 2.36 (s, 3H); ^13^C NMR (acetone-*d_6_*) *δ* 184.1, 182.2, 159.8, 156.1, 138.7, 131.3 (2C), 129.1 (2C), 128.8, 128.5, 106.5, 61.7, 57.0, 21.3; IR *υ*_max_/cm^−1^: 1643 (C=O), 1593 (C=C), 1329 (C–O); LRMS (ESI^+^) *m/z*: 259 (M+H), HRMS (ESI^+^) *m/z* calculated for C_15_H_15_O_4_ (M+H) 259.0970; found 259.0967.

2,5-Dimethoxy-3-m-tolylcyclohexa-2,5-diene-1,4-dione (**14**).

Synthesized according to the general procedure 1B from **10** (0.17 g, 0.67 mmol) and m-tolylboronic acid (0.11 g, 0.80 mmol) under reflux for 72 h. The title compound was obtained as a yellow/orange solid. Yield 0.052 g (30%); m.p. 74–77°C.

^1^H NMR (400 MHz, CDCl_3_) *δ* 7.30 (t, *J* = 7.9 Hz, 1H), 7.19 (d, *J* = 7.5 Hz, 1H), 7.06 (d, *J* = 7.2 Hz, 2H), 5.89 (s, 1H), 3.85 (s, 3H), 3.78 (s, 3H), 2.38 (s, 3H); ^13^C NMR (101 MHz, CDCl_3_) *δ* 183.6, 181.9, 159.0, 155.4, 137.7, 131.3, 130.2, 129.6, 128.0, 127.7, 126.6, 105.7, 61.7, 56.7, 21.6; IR *υ*_max_/cm^−1^: 2852, 1664, 1634, 1581, 1443, 780; LRMS (ESI^+^): *m/z*: 259 (C_15_H_15_O_4_) [M + H].

3-(4-Butylphenyl)-2,5-dimethoxycyclohexa-2,5-diene-1,4-dione (**15**).

Synthesized according to the general procedure 1A from (4-butylphenyl)boronic acid (0.21 g, 1.20 mmol). The title compound was obtained as a yellow solid. Yield 0.26 g (71%); m.p. 130–132°C.

^1^H NMR (acetone-*d_6_*) *δ* 7.24 (dd, *J* = 7.72, 2.2 Hz, 2H), 7.20 (dd, *J* = 7.72, 2.2 Hz, 2H), 6.00 (s, 1H), 3.87 (s, 3H), 3.85 (s, 3H), 2.65 (t, *J* = 7.6 Hz, 2H), 1.67–1.58 (m, 2H), 1.43–1.34 (m, 2H), 0.94 (t, *J* = 7.2 Hz, 3H); ^13^C NMR (acetone-*d_6_*) *δ* 184.2, 182.2, 159.8, 156.1, 143.7, 131.4, 129.0, 128.52 (2C), 128.46 (2C), 106.5, 61.7, 57.0, 36.0, 34.4, 23.1, 14.2; IR *υ*_max_/cm^−1^: 1644 (C=O), 1591 (C=C), 1332 (C–O); LRMS (ESI^+^) *m/z*: 301 (M+H), HRMS (ESI^+^) *m/z* calculated for C_18_H_21_O_4_ (M+H) 300.1440; found 301.1428.

3-(4-Hydroxyphenyl)-2,5-dimethoxycyclohexa-2,5-diene-1,4-dione (**16**).

Synthesized according to the general procedure 1A from 4-hydroxyphenylboronic acid (0.16 g, 1.20 mmol). The title compound was obtained as an orange solid. Yield 0.17 g (68%); m.p. 165–167°C.

^1^H NMR (acetone-*d_6_*) ^1^H NMR (400 MHz, acetone-*d_6_*) *δ* 7.16 (d, *J* = 8.8 Hz, 2H), 6.87 (d, *J* = 8.8 Hz, 12H), 5.97 (s, 1H), 3.87 (s, 3H), 3.83 (s, 3H); ^13^C NMR (acetone-*d_6_*) *δ* 184.2, 182.4, 159.8, 158.4, 155.8, 132.9 (2C), 128.6, 122.5 (2C), 115.4, 106.5, 61.5, 56.9; IR *υ*_max_/cm^−1^: 3330 (O–H), 1631 (C=O), 1593 (C=C), 1300 (C–O); LRMS (ESI^+^) *m/z*: 261 (M+H), HRMS (ESI^+^) *m/z* calculated for C_14_H_13_O_5_ (M+H) 261.0762; found 261.0758.

3-(4-(Hydroxymethyl)phenyl)-2,5-dimethoxycyclohexa-2,5-diene-1,4-diene (**17**).

Synthesized according to the general procedure 1A from 4-hydroxymethylphenylboronic acid (0.18 g, 1.20 mmol). The title compound was obtained as an orange solid. Yield 0.15 g (62%); m.p. 116–118°C.

^1^H NMR (acetone-*d_6_*) *δ* 7.39 (d, *J* = 8.2 Hz, 2H), 7.25 (d, *J* = 8.2 Hz, 2H), 6.00 (s, 1H), 4.66 (s, 2H), 4.25–4.19 (s, br, OH) 3.86 (s, 3H), 3.85 (s, 3H); ^13^C NMR (acetone-*d_6_*): *δ* 184.1, 182.2, 159.8, 156.2, 143.5, 131.2 (2C), 130.3, 128.5, 126.6 (2C), 106.6, 64.5, 61.7, 57.0; IR (KBr) *υ*_max_/cm^−1^: 3249 (OH), 1644 (C=O), 1590 (C=C), 1332 (C–O); LRMS (ESI^+^) *m/z*: 275 (M+H), HRMS (ESI^+^) *m/z* calculated for C_15_H_15_O_5_ (M+H) 275.0919; found 275.0911.

3-(2-Methoxyphenyl)-2,5-dimethoxycyclohexa-2,5-diene-1,4-diene (**18**).

Synthesized according to the general procedure 1B from **10** (0.15 g, 0.58 mmol) and 2-methoxyphenylboronic acid (0.11 g, 0.70 mmol) under reflux for 72 h. The title compound was obtained as a yellow/orange solid. Yield 0.053 g (33%); m.p. 162–164°C.

^1^H NMR (400 MHz, CDCl_3_) *δ* 7.41–7.35 (m, 1H), 7.10 (dd, *J* = 7.5, 1.7 Hz, 1H), 6.98 (ddd, *J* = 14.5, 10.5, 4.6 Hz, 2H), 5.89 (s, 1H), 3.84 (s, 3H), 3.78 (s, 3H), 3.70 (s, 3H); ^13^C NMR (101 MHz, CDCl_3_) *δ* 183.4, 181.2, 159.3, 157.5, 155.8, 131.8, 130.5, 122.7, 120.4, 120.1, 111.1, 105.6, 60.9, 56.6, 55.8; IR *υ*_max_/cm^−1^: 3004, 2952, 2853, 1656, 1589, 1492, 1437, 1016; LRMS (ESI^+^) *m/z*: 275 (M+H), HRMS (ESI^+^) *m/z* calculated for C_15_H_15_O_5_ (M+H) 275.0919; found 275.0931.

3-(3-Methoxyphenyl)-2,5-dimethoxycyclohexa-2,5-diene-1,4-diene (**19**).

Synthesized according to the general procedure 1B from **10** (0.15 g, 0.62 mmol) and 3-methoxyphenylboronic acid (0.11 g, 0.75 mmol) under reflux for 72 h. The title compound was obtained as an orange crystalline solid. Yield 0.088 g (48%); m.p. 72–75°C.

^1^H NMR (400 MHz, CDCl_3_) *δ* 7.32 (t, *J* = 8.0 Hz, 1H), 6.95–6.91 (m, 1H), 6.85 (d, *J* = 7.6 Hz, 1H), 6.83–6.80 (m, 1H), 5.90 (s, 1H), 3.85 (s, 3H), 3.81 (s, 3H), 3.80 (s, 3H); ^13^C NMR (101 MHz, CDCl_3_) *δ* 183.4, 181.7, 159.2, 159.0, 155.4, 131.5, 129.1, 126.1, 123.2, 116.3, 114.5, 105.7, 61.7, 56.7, 55.4; IR *υ*_max_/cm^−1^: 3061, 2943, 2847, 1645, 1591, 1483, 1433, 1213, 970; LRMS (ESI^+^) *m/z*: 275 (M+H), HRMS (ESI^+^) *m/z* calculated for C_15_H_15_O_5_ (M+H) 275.0919; found 275.0925.

2,5-Dimethoxy-3-(2,4-dimethoxyphenyl)cyclohexa-2,5-diene-1,4-dione (**20**).

Synthesized according to the general procedure 1A from 2,4-dimethoxyphenylboronic acid (0.22 g, 1.20 mmol). The title compound was obtained as an orange solid. Yield 0.22 g (75%); m.p 190–192°C.

^1^H NMR (acetone-*d_6_*) *δ* 7.01 (d, *J* = 8.4 Hz, 1H), 6.61 (d, *J* = 2.4 Hz, 1H), 6.56 (dd, *J* = 8.4, 2.4 Hz, 1H), 5.97(s, 1H), 3.87 (s, 3H), 3.84 (s, 3H), 3.75 (s, 6H); ^13^C NMR (acetone-*d_6_*) *δ* 183.9, 181.7, 162.5, 160.1, 159.4, 156.5, 132.9, 125.3, 113.6, 106.4, 105.4, 99.1, 60.9, 56.9, 55.9, 55.7; IR *υ*_max_/cm^−1^: 1649 (C=O), 1575 (C=C), 1329 (C–O); LRMS (ESI^+^) *m/z*: 305 (M+H), HRMS (ESI^+^) *m/z* calculated for C_16_H_17_O_6_ (M+H) 305.1024; found 305.1020.

2,5-Dimethoxy-3-(2,5-dimethoxyphenyl)cyclohexa-2,5-diene-1,4-dione (**21**).

Synthesized according to the general procedure 1A from 2,5-dimethoxyphenylboronic acid (0.22 g, 1.20 mmol). The title compound was obtained as an orange solid. Yield 0.22 g (74%); m.p. 180–182°C.

^1^H NMR (acetone-*d_6_*) *δ* 6.98 (d, *J* = 8.6 Hz, 1H), 6.93 (dd, *J* = 8.6, 2.8 Hz, 1H), 6.73 (d, *J* = 2.8 Hz, 1H), 5.99 (s, 1H), 3.87 (s, 3H), 3.78 (s, 3H), 3.75 (s, 3H), 3.71 (s, 3H); ^13^C NMR (acetone-*d*_6_) *δ* 183.9, 181.7, 162.5, 160.1, 159.4, 156.5, 132.9, 125.3, 113.6, 106.4, 105.4, 99.1, 60.9, 56.9, 55.9, 55.7; IR *υ*_max_/cm^−1^: 1644 (C=O), 1587 (C=C), 1326 (C–O); LRMS (ESI^+^) *m/z*: 305 (M+H), HRMS (ESI^+^) *m/z* calculated for C_16_H_17_O_6_ (M+H) 305.1024; found 305.1020.

2,5-Dimethoxy-3-(2,3-dimethoxyphenyl)cyclohexa-2,5-diene-1,4-dione (**22**).

Synthesized according to the general procedure 1B from **10** (0.16 g, 0.64 mmol) and 2,3-dimethoxyphenylboronic acid (0.14 g, 0.76 mmol) under reflux for 72 h. The title compound was obtained as a yellow/orange solid. Yield 0.050 g (26%); m.p. 122–125°C.

^1^H NMR (400 MHz, CDCl_3_) *δ* 7.07 (t, *J* = 7.9 Hz, 1H), 6.97 (dd, *J* = 8.2, 1.4 Hz, 1H), 6.69 (dd, *J* = 7.6, 1.5 Hz, 1H), 5.90 (s, 1H), 3.89 (s, 3H), 3.85 (s, 3H), 3.77 (s, 3H), 3.71 (s, 3H); ^13^C NMR (101 MHz, CDCl_3_) *δ* 183.2, 181.6, 159.2, 155.8, 152.7, 147.3, 125.2, 123.7, 123.0, 122.7, 113.3, 105.7, 61.0, 60.9, 56.7, 55.8; IR *υ*_max_/cm^−1^: 2852, 1664, 1634, 1581, 1443, 780; LRMS (ESI^+^) *m/z*: 305 (M + H), HRMS (ESI^+^) *m/z* calculated for C_16_H_17_O_6_ (M+H) 305.1024; found 305.1030.

2,5-Dimethoxy-3-(3,4-dimethoxyphenyl)cyclohexa-2,5-diene-1,4-dione (**23**).

Synthesized according to the general procedure 1B from **10** (0.16 g, 0.65 mmol) and 3,4-dimethoxyphenylboronic acid (0.14 g, 0.78 mmol) under reflux for 72 h. The title compound was obtained as a yellow/orange solid. Yield 0.042 g (22%); m.p. 135–137°C.

^1^H NMR (400 MHz, CDCl_3_) *δ* 6.93–6.87 (m, 2H), 6.83 (d, *J* = 1.7 Hz, 1H), 5.89 (s, 1H), 3.91 (s, 3H), 3.86 (d, *J* = 4.9 Hz, 6H), 3.80 (s, 3H); ^13^C NMR (101 MHz, CDCl_3_) *δ* 183.5, 182.1, 158.9, 155.3, 149.5, 148.5, 126.3, 123.7, 122.4, 114.0, 110.7, 105.7, 61.5, 56.7, 56.1, 56.0; IR *υ*_max_/cm^−1^: 2837, 1671, 1633, 1592, 1434, 796; LRMS (ESI^+^) *m/z*: 305 (M + H), HRMS (ESI^+^) *m/z* calculated for C_16_H_17_O_6_ (M + H) 305.1024; found 305.1011.

2,5-Dimethoxy-3-(naphthalene-3-yl)cycloheza-2,5-diene-1,4-dione (**24**).

Synthesized according to the general procedure 1A from 2-naphthylboronic acid (0.20 g, 1.20 mmol). The title compound was obtained as a yellow solid. Yield 0.20 g (70%). m.p. 137–139°C.

^1^H NMR (acetone-*d_6_*) *δ* 7.94–7.90 (m, 3H), 7.84 (s, 1H), 7.57–7.50 (m, 2H), 7.42 (dd, *J* = 8.2, 1.6 Hz, 1H), 6.05 (s, 1H), 3.90 (s, 3H), 3.88 (s, 3H); ^13^C NMR (acetone-*d_6_*) *δ* 184.1, 182.1, 159.8, 156.4, 133.9, 133.8, 130.9, 129.5, 129.2, 129.0, 128.5, 128.2, 127.8, 127.3, 127.0, 106.6, 61.8, 57.0; IR *υ*_max_/cm^−1^: 1644 (C=O), 1583 (C=C), 1293 (C–O); MS (ESI^+^) *m/z*: 295 (M+H), HRMS (ESI^+^) *m/z* calculated for C_18_H_15_O_4_ (M+H) 295.0970; found 295.0965.

2,5-dimethoxy-3-(naphthalene-1-yl)cyclohexa-2,5-diene-1,4-dione (**25**).

Synthesized according to the general procedure 1A from 1-naphthylboronic acid (0.20 g, 1.20 mmol). The title compound was obtained as a yellow solid. Yield 0.19 g (65%); m.p. 134–137°C.

^1^H NMR (acetone-*d_6_*) *δ* 7.95 (d, *J* = 8.0 Hz, 2H), 7.74 (d, *J* = 8.0 Hz, 1H), 7.57–7.46 (m, 3H), 7.38 (dd, *J* = 8.0, 1.2 Hz, 1H), 6.10 (s, 1H), 3.90 (s, 3H), 3.67 (s, 3H); ^13^C NMR (acetone-*d_6_*) *δ* 183.2, 181.3, 158.9, 155.5, 133.0, 132.9, 129.9, 128.6, 128.3, 128.1, 127.6, 127.3, 126.9, 126.4, 126.1, 105.8, 60.9, 56.2; IR *υ*_max_/cm^−1^: 1650 (C=O), 1587 (C=C), 1331 (C–O); LRMS (ESI^+^) *m/z*: 295 (M+H), HRMS (ESI^+^) *m/z* calculated for C_18_H_15_O_4_ (M+H) 295.0970; found 295.0965.

3-(Benzo[b]thiophen-2-yl)-2,5-dimethoxycyclohexa-2,5-diene-1,4-dione (**26**).

Synthesized according to the general procedure 1A from 2-benzothienylboronic acid (0.21 g, 1.20 mmol). The title compound was obtained as a red solid. Yield 0.19 g (64%); m.p. 205–207°C.

^1^H NMR (acetone-*d_6_*) *δ* 8.27 (s, 1H), 7.96–7.90 (m, 2H), 7.41–7.37 (m, 2H), 6.04 (s, 1H), 4.24 (s, 3H), 3.92 (s, 3H); ^13^C NMR (acetone-*d_6_*) *δ* 183.3, 181.4, 159.9, 155.6, 141.8, 139.9, 132.4, 129.2, 126.2, 125.2, 125.1, 122.4, 121.5, 106.5, 62.0, 57.3; IR *υ*_max_/cm^−1^: 1667 (C=O), 1563 (C=C), 1208 (C–O); LRMS (ESI^+^) *m/z*: 301 (M + H), HRMS (ESI^+^) *m/z* calculated for C_16_H_13_O_4_S (M+H) 301.0534; found 301.0528.

3-Biphenyl-4-yl-2,5-dimethoxycyclohexa-2,5-diene-1,4-dione (**27**).

Synthesized according to the general procedure 1A from 4-biphenylboronic acid (0.21 g, 1.20 mmol). The title compound was obtained as a red solid with identical spectral data to those previously reported [30]. Yield 0.22 g (69%); m.p. 178–180°C (Lit. 182–184°C) [31].

^1^H NMR (acetone-*d_6_*) *δ* 7.73–7.69 (m, 4H), 7.50–7.46 (m, 2H), 7.42–7.36 (m, 3H), 6.03 (s, 1H), 3.92 (s, 3H), 3.89 (s, 3H); ^13^C NMR (acetone-*d*_6_) *δ* 184.1, 182.1, 159.8, 156.3, 141.5, 141.4, 132.0 (2C), 130.9, 129.8 (2C), 128.4, 128.1, 127.8 (2C), 126.9 (2C), 106.6, 61.8, 57.0; IR *υ*_max_/cm^−1^: 1669 (C=O), 1582 (C=C), 1325 (C–O); LRMS (ESI^+^) *m/z*: 321 (M + H), HRMS (ESI^+^) *m/z* calculated for C_20_H_17_O_4_ (M+H) 321.1127; found 321.1120.

3-Biphenyl-3-yl-2,5-dimethoxycyclohexa-2,5-diene-1,4-dione (**28**).

Synthesised according to the general procedure 1A from 3-biphenylboronic acid (0.21 g, 1.20 mmol). The title compound was obtained as a red solid. Yield 0.21 g (68%); m.p. 172–174°C.

^1^H NMR (acetone-*d*_6_) *δ* 7.69–7.64 (m, 3H), 7.59 (t, *J* = 1.6 Hz, 1H), 7.53–7.45 (m, 3H), 7.37 (t, *J* = 8.0 Hz, 1H), 7.30 (d, *J* = 8.0 Hz, 1H), 6.03 (s, 1H), 3.92 (s, 3H), 3.89 (s, 3H); ^13^C NMR (acetone-*d_6_*) *δ* 184.1, 182.1, 159.9, 156.3, 141.6, 141.3, 132.5, 130.4, 130.0 (2C), 129.8, 129.1, 128.3, 127.8 (2C), 127.5 (2C), 106.6, 61.8, 57.0; IR *υ*_max_/cm^−1^: 1670 (C=O), 1581 (C=C), 1336 (C–O); LRMS (ESI^+^) *m/z*: 321 (M+H), HRMS (ESI^+^) *m/z* calculated for C_20_H_17_O_4_ (M+H) 321.1127; found 321.1120.

3-Biphenyl-2-yl-2,5-dimethoxycyclohexa-2,5-diene-1,4-dione (**29**).

Synthesized according to the general procedure 1A from 2-biphenylboronic acid (0.21 g, 1.20 mmol). The title compound was obtained as a red solid. Yield 0.21 g (67%); m.p. 186–188°C.

^1^H NMR (acetone-*d*_6_) *δ* 7.47 (td, *J* = 8.0, 1.6 Hz, 1H), 7.41 (td, *J* = 8.0, 1.6 Hz, 2H), 7.35–7.31 (m, 2H), 7.29–7.22 (m, 4H), 5.89 (s, 1H), 3.81 (s, 3H), 3.68 (s, 3H); ^13^C NMR (acetone-*d_6_*) *δ* 183.9, 181.9, 159.7, 156.0, 143.2, 142.4, 131.9, 130.9, 130.3, 129.4, 129.3 (2C), 128.9 (2C), 128.6, 128.0, 127.6, 106.5, 61.2, 57.0; IR *υ*_max_/cm^−1^: 1646 (C=O), 1589 (C=C), 1325 (C–O); LRMS (ESI^+^) *m/z*: 321 (M+H), HRMS (ESI^+^) *m/z* calculated for C_20_H_17_O_4_ (M+H) 321.1127; found 321.1121.

General procedure 2 for synthesis of symmetrical 2,5-dimethoxy-3,6-diaryl-2,5-diene-1,4-diones by Suzuki coupling.

A Biotage microwave vial was filled with **11** (0.32 g, 1.00 mmol), relevant boronic acid (2.40 mmol), K_2_CO_3_ (0.34 g, 2.50 mmol) and Pd(dppf)Cl_2_ (0.07 g, 0.01 mmol), and then dioxane (5 ml) was added to this mixture. The reaction vessel was sealed and irradiated with microwaves at 120°C for 20 min (holding time) with a magnetic bar stirrer. After cooling with compressed air to 40°C, the reaction mixture was diluted with dioxane (10 ml) and filtered through the Celite^®^. The reaction crude was dried *in vacuo* and subjected to column chromatography for purification (10% EtOAc-90% hexane) to afford an orange-red solid.

2,5-Dimethoxy-3,6-diphenylcyclohexa-2,5-diene-1,4-dione (**30**).

Synthesized according to the general procedure 2 from phenylboronic acid (0.29 g, 2.40 mmol). The title compound was obtained as a yellow solid with identical spectral data to those of the previous report [31]. Yield 0.22 g (70%); m.p. 150–152°C (Lit. 148–150°C) [31].

^1^H NMR (CDCl_3_): *δ* 7.47–7.40 (m, 6H), 7.38–7.34 (m, 4H), 3.83 (s, 6H); ^13^C NMR (CDCl_3_): *δ* 183.6 (2C), 154.8 (2C), 130.7 (4C), 130.3 (2C), 128.7 (2C), 128.1 (4C), 126.7 (2C), 61.7 (2C); IR *υ*_max_/cm^−1^: 1648 (C=O), 1592(C=C), 1295 (C–O); LRMS (ESI^+^) *m/z*: 321 (M + H), HRMS (ESI^+^) *m/z* calculated for C_20_H_17_O_4_ (M + H) 321.1126; found 321.1118.

2,5-Bis(4-butylphenyl)-3,6-dimethoxycyclohexa-2,5-diene-1,4-dione (**31**).

Synthesized according to the general procedure 2 from 4-butylphenylboronic acid (0.39 g, 2.40 mmol). The title compound was obtained as an orange solid. Yield 0.27 g (65%); m.p. 177–179°C.

^1^H NMR (acetone-*d_6_*) *δ* 7.30–7.25 (m, 8H), 3.85 (s, 6H), 3.77 (t, *J* = 7.6 Hz, 4H), 1.65 (m, 4H), 1.40 (m, 4H), 0.95 (t, *J* = 7.2 Hz, 6H); ^13^C NMR (acetone-*d_6_*) *δ* 184.3 (2C), 155.7 (2C), 143.8 (2C), 131.5 (4C), 129.0 (2C), 128.8 (2C), 128.5 (4C), 61.6 (2C), 36.1 (2C), 34.4 (2C), 23.1 (2C), 14.2 (2C); IR *υ*_max_/cm^−1^: 1647 (C=O), 1583 (C=C), 1301 (C–O); LRMS (ESI^+^) *m/z*: 433 (M+H), HRMS (ESI^+^) *m/z* calculated for C_28_H_33_O_4_ (M+H) 433.2378; found 433.2370.

General procedure 3 for synthesis of asymmetrical 2,5-dimethoxy-3,6-diaryl-2,5-diene-1,4-diones by Suzuki coupling.

A 5 ml Biotage microwave vial was filled with **11** (0.32 g, 1.00 mmol), 1-naphthylboronic acid or 2-naphthylboronic acid (0.20 g, 1.20 mmol), K_2_CO_3_ (0.34 g, 2.50 mmol) and Pd(dppf)Cl_2_ (0.07 g, 0.01 mmol), and then dioxane (5 ml) was added to this mixture. The reaction vessel was sealed and irradiated with microwaves at 120°C for 20 min (holding time) with a magnetic bar stirrer. After cooling with compressed air to 40°C, the second relevant boronic acid (1.20 mmol) with a similar portion of catalyst was added to the reaction crude, and irradiation was continued for further 20 min at 120°C. The microwave vial was cooled with compressed air and then the mixture was diluted with dioxane (10 ml) and filtered through the Celite®. The reaction crude was dried *in vacuo* and subjected to column chromatography for purification (10% EtOAc-90% hexane) to afford an orange-red solid.

2,5-Dimethoxy-3-(naphthalen-1-yl)-6-phenylcyclohexa-2,5-diene-1,4-dione (**32**).

Synthesized according to the general procedure 3 from 1-naphthylboronic acid (0.20 g, 1.20 mmol) and phenylboronic acid (0.14 g, 1.20 mmol). The title compound was obtained as an orange solid. Yield 0.25 g (70%); m.p. 168–170°C.

^1^H NMR (acetone-*d_6_*) *δ* 7.99–7.97 (m, 2H), 7.58 (dd, *J* = 8.0, 1.2 Hz, 1H), 7.59 (t, *J* = 8.0, 1H), 7.56–7.48 (m, 2H), 7.46–7.40 (m, 5H), 3.85 (s, 3H), 3.68 (s, 3H); ^13^C NMR NMR (acetone-*d_6_*) *δ* 184.2, 183.8, 156.9, 156.0, 134.4, 133.3, 132.0, 131.6 (2C), 130.4, 129.5, 129.24, 129.20, 129.0, 128.9, 128.5 (2C), 127.1, 126.74, 126.69, 126.2, 126.0, 61.7, 61.1; IR *υ*_max_/cm^−1^: 1647 (C=O), 1587 (C=C), 1297 (C–O); LRMS (ESI^+^) *m/z*: 371 (M+H), HRMS (ESI^+^) *m/z* calculated for C_24_H_19_O_4_ (M+H) 371.1283; found 371.1278.

2,5-Dimethoxy-3-(naphthalene-1-yl)-6-(naphthalene-3-yl)cyclohexa-2,5-diene-2,5-diene-1,4-dione (**33**).

Synthesized according to the general procedure 3 from 1-naphthylboronic acid (0.20 g, 1.20 mmol) and 2-naphthyl boronic acid (0.20 g, 1.20 mmol). The title compound was obtained as an orange solid. Yield 0.27 g (66%); m.p. 163–165°C.

^1^H NMR (acetone-*d_6_*) *δ* 8.00–7.94 (m, 6H), 7.89 (dd, *J* = 8.0, 1.2 Hz, 1H), 7.61–7.51 (m, 6H), 7.47 (dd, *J* = 8.0, 1.2 Hz, 1H), 3.88 (s, 3H), 3.69 (s, 3H); ^13^C NMR (acetone-*d_6_*) *δ* 184.3, 183.9, 156.9, 156.3, 134.5, 134.0, 133.8, 133.3, 131.1, 130.5, 129.6, 129.5, 129.3, 129.28, 129.27, 129.1, 128.7, 128.5, 127.8, 127.4, 127.12, 127.05, 126.8, 126.7, 126.3, 126.0, 61.8, 61.1; IR *υ*_max_/cm^−1^: 1645 (C=O), 1586 (C=C), 1299 (C–O); LRMS (ESI^+^) *m/z*: 421 (M + H), HRMS (ESI^+^) *m/z* calculated for C_28_H_21_O_4_ (M+H) 421.1440; found 421.1434.

2,5-Dimethoxy-3-(2,4-dimethoxyphenyl)-6-(naphthalene-3-yl)cyclohexa-2,5-diene-1,4-dione (**34**).

Synthesized according to the general procedure 3 from 2-naphthyl boronic acid (0.20 g, 1.20 mmol) and 2,4-dimethoxyphenyl boronic acid (0.21 g, 1.00 mmol). The title compound was obtained as an orange solid. Yield 0.29 g (69%); m.p. 159–161°C.

^1^H NMR (acetone-*d_6_*) *δ* 7.95–7.92 (m, 4H), 7.67–7.54 (m, 2H), 7.50 (dd, *J* = 8.4, 2.4 Hz, 1H), 7.14 (d, *J* = 8.0 Hz, 1H), 6.66 (d, *J* = 2.4 Hz, 1H), 6.61 (dd, *J* = 8.4, 2.4 Hz, 1H), 3.88 (s, 3H), 3.86 (s, 3H), 3.82 (s, 3H), 3.80 (s, 3H); ^13^C NMR (acetone-*d_6_*) *δ* 184.0, 183.7, 162.6, 159.5, 156.5, 156.0, 134.0, 133.8, 133.0, 131.0, 129.5, 129.3, 129.1, 128.5, 128.1, 127.7, 127.4, 127.0, 125.8, 113.5, 105.5, 99.2, 61.7, 61.1, 56.0, 55.7; IR *υ*_max_/cm^−1^: 1665 (C=O), 1606 (C=C), 1300 (C–O); LRMS (ESI^+^) *m/z*: 431 (M+H), HRMS (ESI^+^) *m/z* calculated for C_26_H_23_O_6_ (M + H) 431.1494; found 431.1489.

2-(4-Butylphenyl)-3,6-dimethoxy-5-(naphthalene-3-yl)cyclohexa-2,5-diene-1,4-dione (**35**).

Synthesized according to the general procedure 3 from 2-naphthyl boronic acid (0.20 g, 1.20 mmol) and 4-butylphenyl boronic acid (0.21 g, 1.20 mmol). The title compound was obtained as an orange solid. Yield 0.28 g (67%); m.p. 134–136°C.

^1^H NMR (acetone-*d_6_*) *δ* 7.96–7.92 (m, 4H), 7.57–7.54 (m, 2H), 7.50 (dd, *J* = 8.2, 1.2 Hz, 1H), 7.33 (d, *J* = 8.2 Hz, 2H), 7.27 (d, *J* = 8.2 Hz, 2H), 3.89 (s, 3H), 3.88 (s, 3H), 2.66 (t, *J* = 7.6 Hz, 2H), 1.66 (m, 2H), 1.43 (m, 2H), 0.94 (t, *J* = 7.2 Hz, 3H); ^13^C NMR (acetone-*d*_6_) *δ* 184.32, 184.27, 156.1, 155.7, 143.9, 134.0, 133.8, 131.5 (2C), 131.0, 129.4, 129.2, 129.1, 129.0, 128.9, 128.52 (2C), 128.48, 127.8 (2C), 127.4, 127.0, 61.8, 61.7, 36.1, 34.4, 23.1, 14.2; IR *υ*_max_/cm^−1^: 1641 (C=O), 1586 (C=C), 1297 (C–O); LRMS (ESI^+^) *m/z*: 427 (M+H), HRMS (ESI^+^) *m/z* calculated for C_28_H_27_O_4_ (M+H) 427.1909; found 427.1901.

2,5-Dimethoxy-3-(biaryl-2-yl)-6-(naphthalene-3-yl)cyclohexa-2,5-diene-1,4-dione (**36**).

Synthesized according to the general procedure 3 from 2-naphthyl boronic acid (0.20 g, 1.20 mmol) and 2-biphenylboronic acid (0.23 g, 1.20 mmol). The title compound was obtained as an orange solid. Yield 0.28 g (65%); m.p. 113–115°C.

^1^H NMR (acetone-*d_6_*) *δ* 7.94–7.90 (m, 3H), 7.83 (d, *J* = 1.6 Hz, 1H), 7.56–7.44 (m, 5H), 7.43–7.30 (m, 7H), 3.83 (s, 3H), 3.56 (s, 3H); ^13^C NMR (acetone-*d_6_*) *δ* 183.9, 183.8, 156.4, 155.8, 143.1, 142.7, 133.9, 133.7, 132.1, 131.0, 130.9 (2C), 130.3, 129.62, 129.56, 129.4, 129.2, 129.1, 129.0 (2C), 128.4 (2C), 128.3, 128.0, 127.72, 127.71, 127.4, 127.0, 61.4, 61.3; IR *υ*_max_/cm^−1^: 1646 (C=O), 1583 (C=C), 1300 (C–O); LRMS (ESI^+^) *m/z*: 447 (M + H), HRMS (ESI^+^) *m/z* calculated for C_30_H_23_O_4_ (M + H) 447.1596; found 447.1592.

2,5-Dimethoxy-3-(biaryl-3-yl)-6-(naphthalene-3-yl)cyclohexa-2,5-diene-1,4-dione (**37**).

Synthesized according to the general procedure 3 from 2-naphthyl boronic acid (0.20 g, 1.20 mmol) and 3-biphenyl boronic acid (0.23 g, 1.20 mmol). The title compound was obtained as an orange solid. Yield 0.28 g (65%); m.p. 119–121°C.

^1^H NMR (CDCl_3_) *δ* 7.92–7.87 (m, 4H), 7.65–7.60 (m, 4H), 7.55–7.51 (m, 3H), 7.48–7.44 (m, 3H), 7.39–7.35 (m, 2H), 3.90 (s, 3H), 3.84 (s, 3H); ^13^C NMR (CDCl_3_) *δ* 183.7, 183.6, 155.1, 155.0, 141.1, 140.9, 133.3, 132.9, 130.8, 130.5, 129.61 (2C), 129.60, 129.0, 128.6, 128.5, 128.1, 127.9, 127.8, 127.62 (2C), 127.59, 127.4 (2C), 126.9, 126.72, 126.67, 126.4, 61.88, 61.87; IR *υ*_max_/cm^−1^: 1647 (C=O), 1583 (C=C), 1301 (C–O); LRMS (ESI^+^) *m/z*: 447 (M+H), HRMS (ESI^+^) *m/z* calculated for C_30_H_23_O_4_ (M+H) 447.1596; found 447.1592.

General procedure 4 for synthesis of aryl-2,5-bisaminocyclohexa-2,5-diene-1,4-dione.

3-Aryl-2,5-dimethoxy-1,4-benzoquinone or 3,5-diaryl-2,5-dimethoxy-1,4-benzoquinone, synthesized following the general procedures 1 and 3 (1.00 mmol), was dissolved in MeOH (20 ml); then the relevant aliphatic amine (2.00 mmol) was added dropwise. The reaction was stirred at room temperature for 1 h, and the precipitate filtered and washed with MeOH (2 × 20 ml). The desired product dried *in vacuo* as a red to pink solid.

2,5-Bis(propylamino)3-p-tolylcyclohexa-2,5-diene-1,4-dione (**38**).

Synthesized according to the general procedure 4 from **13** (0.25 g, 1.00 mmol) and *n*-propyl amine (0.17 ml, 2.00 mmol). The title compound was obtained as a pink solid. Yield 0.27 g (95%); m.p. 166–168°C.

^1^H NMR (DMSO-*d_6_*) *δ* 7.75–7.72 (s, br, NH), 7.36–7.33 (s, br, NH), 7.15 (d, *J* = 7.6 Hz, 2H), 7.04 (d, *J* = 7.60 Hz, 2H), 5.34 (s, 1H), 3.15–3.09 (m, 2H), 2.56–2.52 (m, 2H), 2.32 (s, 3H), 1.58–1.53 (m, 2H), 1.24–1.18 (m, 2H), 0.88 (t, *J* = 7.6 Hz, 3H), 0.47 (t, *J* = 7.6 Hz, 3H); ^13^C NMR (DMSO-*d_6_*) *δ* 177.8, 177.2, 150.9, 146.6, 135.8, 132.1, 131.3, 128.1 (2C), 106.8 (2C), 91.4, 45.0, 43.5, 22.3, 20.9, 20.8, 11.3, 10.7; IR *υ*_max_/cm^−1^: 3243 (–NH), 1643 (C=O), 1586 (C=C), 1347 (C–O); LRMS (ESI^+^) *m/z*: 313 (M + H), HRMS (ESI^+^) *m/z* calculated for C_19_H_24_N_2_O_2_ (M + H) 313.1915; found 313.1910.

3-(Naphthalene-1-yl)-2,5-bis-(propylamino)cyclohexa-2,5-diene-1,4-dione (**39**).

Synthesized according to the general procedure 4 from **25** (0.30 g, 1.00 mmol) and *n*-propyl amine (0.17 ml, 2.00 mmol). The title compound was obtained as a pink solid. Yield 0.21 g (85%); m.p. 134–137°C.

^1^H NMR (DMSO-*d_6_*) *δ* 7.95 (d, *J* = 7.6 Hz, 1H), 7.91 (d, *J* = 8.4 Hz, 1H), 7.76–7.73 (m, NH), 7.66 (d, *J* = 7.6 Hz, 1H), 7.53–7.44 (m, 4H), 7.34 (dd, *J* = 7.0, 1.0 Hz, 1H), 5.45 (s, 1H), 3.18–3.13 (m, 2H), 2.21–2.14 (m, 2H), 1.63–1.54 (m, 2H), 1.07–0.93 (m, 2H), 0.90 (t, *J* = 7.6 Hz, 3H), 0.16 (t, J = 7.6 Hz, 3H); ^13^C NMR (DMSO-d_6_) *δ* 177.4, 177.1, 151.1, 147.3, 133.2, 132.94, 132.89, 129.1, 128.2, 127.5, 126.1, 125.7 (2C), 125.3, 104.0, 91.7, 44.4, 43.6, 22.7, 21.0, 114, 10.4; IR *υ*_max_/cm^−1^: 3263 (–NH), 1643 (C=O), 1581 (C=C), 1346 (C–O); LRMS (ESI^+^) *m/z*: 349 (M + H), HRMS (ESI^+^) *m/z* calculated for C_22_H_25_N_2_O_2_ (M + H) 349.1916; found 349.1909.

2,5-Bis(3-(dimethylamino)propylamino)-3-(naphthalene-1-yl)cyclohexa-2,5-diene-1,4-diene (**40**).

Synthesized according to the general procedure 4 from **25** (0.30 g, 1.00 mmol) and *N,N*-dimethylpropane-1,3-diamine (0.20 g, 2.00 mmol). The title compound was obtained as a red solid. Yield: 0.17 g (84%); m.p. 210–212°C.

^1^H NMR (DMSO-*d_6_*) *δ* 8.0 (s, br, NH), 7.94 (d, *J* = 7.6 Hz, 1H), 7.90 (d, *J* = 7.6 Hz, 1H), 7.84 (s, br, NH), 7.67 (d, *J* = 7.6 Hz, 1H), 7.52–7.44 (m, 3H), 7.34 (d, 7.6 Hz, 1H), 5.43 (s, 1H), 3.25–3.20 (m, 2H), 2.36–2.26 (m, 4H), 2.12 (s, 6H), 1.83 (s, 6H), 1.73–1.66 (m, 2H), 1.60–1.48 (m, 2H), 1.16–1.00 (m, 2H); ^13^C NMR (DMSO-d_6_) *δ* 177.4, 177.2, 151.1, 147.3, 133.2, 133.0, 132.9, 129.1, 128.2, 127.5, 126.1, 125.7 (2C), 125.3, 104.0, 91.7, 44.4, 43.6, 30.7 (4C), 22.7, 21.0, 11.4, 10.4; IR *υ*_max_/cm^−1^: 3271 (–NH), 1629 (C=O), 1569 (C=C), 1332 (C–O); LRMS (ESI^+^) *m/z*: 435 (M + H), HRMS (ESI^+^) *m/z* calculated for C_26_H_35_N_4_O_2_ (M + H) 435.2759; found 435.2752.

3-(Biaryl-2-yl)-2,5-bis-(propylamino)cyclohexa-2,5-diene-1,4-dione (**41**).

Synthesized according to the general procedure 4 from **29** (0.30 g, 1.00 mmol) and *n*-propyl amine (0.17 ml, 2.00 mmol). The title compound was obtained as a red solid. Yield 0.31 g (85%); m.p. 177–179°C.

^1^H NMR (acetone-*d_6_*) *δ* 7.44–7.40 (m, 1H), 7.38–7.33 (m, 2H), 7.31–7.21 (m, 6H), 7.04 (bs, NH), 6.76 (bs, NH), 5.25 (s, 1H), 3.21–3.16 (m, 2H), 2.71–2.56 (m, 2H), 1.71–1.62 (m, 2H), 1.34–1.30 (m, 2H), 0.95 (t, *J* = 7.2 Hz, 3H), 0.64 (t, *J* = 7.2 Hz, 3H); ^13^C NMR (acetone-*d_6_*) *δ* 178.8, 151.5, 144.4, 142.8, 134.8, 133.8, 130.1, 129.3, 128.7, 128.6 (2C), 127.7, 127.5, 92.5, 46.0, 45.8, 44.7, 44.5, 22.78, 23.75, 22.15, 22.13, 11.6, 11.2; IR *υ*_max_/cm^−1^: 3272 (–NH), 1652 (C=O), 1587 (C=C), 1295 (C–O); LRMS (ESI^+^) *m/z*: 375 (M + H), HRMS (ESI^+^) *m/z* calculated for C_24_H_27_N_2_O_2_ (M + H) 375.2072; found 375.2067.

2-(Naphthalene-1-yl)-5-phenyl-3,6-bis-(propylamino)cyclohexa-2,5-diene-1,4-dione (**42**).

Synthesized according to the general procedure 4 from **32** (0.37 g, 1.00 mmol) and *n*-propyl amine (0.17 ml, 2.00 mmol). The title compound was obtained as a red solid. Yield 0.37 g (88%); m.p. 226–228°C.

^1^H NMR (DMSO-*d_6_*) *δ* 7.98–7.92 (m, 2H), 7.76–7.74 (m, 1H), 7.56–7.50 (m, 4H), 7.44–7.29 (m, 7H), 2.59–2.54 (m, 2H), 2.26–2.18 (m, 2H), 1.29–1.20 (m, 2H), 1.09–0.94 (m, 2H), 0.46 (t, *J* = 7.2 Hz, 3H), 0.17 (t, *J* = 7.2 Hz, 3H); ^13^C NMR (DMSO-*d_6_*) *δ* 177.7 (2C), 177.4 (2C), 147.0 (2C), 146.3, 135.2, 133.1, 133.0, 131.6, 129.1, 128.3, 127.6, 126.9, 126.2 (2C), 125.8 (2C), 125.3, 106.6, 103.5, 45.2, 44.5, 22.8, 22,4, 10.7, 10.5; IR *υ*_max_/cm^−1^: 3245 (–NH), 1640 (C=O), 1560 (C=C), 1303 (C–O); LRMS (ESI^+^) *m/z*: 425 (M + H), HRMS (ESI^+^) *m/z* calculated for C_28_H_29_N_2_O_2_ (M + H) 425.2228; found 425.2221.

2-(Naphthalene-1-yl)-5-naphthalen-3-yl)-3,6-bis(propylamino)cyclohexa-2,5-diene-1,4-dione (**43**).

Synthesized according to the general procedure 4 from **33** (0.42 g, 1.00 mmol) and *n*-propyl amine (0.17 ml, 2.00 mmol). The title compound was obtained as a red solid. Yield 0.79 g (88%); m.p. 210–213°C.

^1^H NMR (DMSO-*d_6_*) *δ* 7.99–7.91 (m, 5H), 7.83 (bs, NH), 7.79–7.77 (m, 1H), 7.58–7.43 (m, 9H), 2.59–2.54 (m, 2H), 2.23–2.20 (m, 2H), 1.27–1.18 (m, 2H), 1.12–0.93 (m, 2H), 0.31 (t, *J* = 7.2, 3H), 0.18 (t, *J* = 7.2, 3H); ^13^C NMR (DMSO-d_6_) *δ* 177.7, 177.6, 147.0, 146.7, 133.1, 132.9, 132.8, 132.6, 131.9 (2C), 130.0 (2C), 129.8, 129.1, 128.3, 127.7, 127.6, 127.5, 126.7, 126.2, 126.1, 125.9, 125.8, 125.3, 106.4, 103.5, 45.3, 44.5, 22.7, 22.2, 10.6, 10.4; IR *υ*_max_/cm^−1^: 3243 (–NH), 1640 (C=O), 1557 (C=C), 1295 (C–O); LRMS (ESI^+^) *m/z*: 475 (M+H), HRMS (ESI^+^) *m/z* calculated for C_32_H_30_N_2_O_2_ (M+H) 475.2385; found 475.2379.

## Supplementary Material

Supporting Data

## Data accessibility

The datasets supporting this article have been uploaded as part of the electronic supplementary material.
