# Supplementary material for: A focused library synthesis and cytotoxicity of quinones derived from the natural product bolinaquinone
Source: R Soc Open Sci. 2018 Apr 4;5(4):171189. doi: 10.1098/rsos.171189 (PMC5936891; doi:10.1098/rsos.171189)

**Focused library synthesis of bolinaquinone analogues as cytotoxic agents by microwave irradiation**

Azadeh Ghods,^a^  Jayne Gilbert,^b^ Jennifer R. Baker, ^a^ Cecilia C. Russell, ^a^ Jennette A. Sakoff ^a,b^ and Adam McCluskey ^a*^

^a^ *Centre for Chemical Biology, Chemistry, School of Environmental and Life Sciences, The University of Newcastle, Callaghan, NSW 2308, Australia. Phone: +61 29 216486; Fax: +61 29 215472; E-mail:* [Adam.McCluskey@newcastle.edu.au](mailto:Adam.McCluskey@newcastle.edu.au)

^b^ *Department of Medical Oncology, Calvary Mater Newcastle Hospital, Waratah, NSW 2298, Australia*

**Supplementary Data**

**Table S1.** Percentage of Cell Growth Inhibition in response to 25 μM of Drug- Library A.

|  | | | | | | | | | | | | |
| --- | --- | --- | --- | --- | --- | --- | --- | --- | --- | --- | --- | --- |
|  | **HT29^a^** | **U87^b^** | **MCF-7^c^** | **A2780^d^** | **H460^e^** | **A431^f^** | **Du145^g^** | **BE2-C^h^** | **SJ-G2^b^** | **MIA^i^** | **SMA^j^** | **MCF10A^k^** |
| **12** | 24 ± 1 | 12 ± 2 | 40 ± 3 | 75 ± 7 | <10 | 10 ± 3 | <10 | >100 | 27 ± 6 | 31 ± 5 | 49 ± 8 | <10 |
| **13** | 10 ± 4 | 13 ± 4 | 23 ± 3 | 72 ± 10 | 13 ± 3 | <10 | 13 ± 2 | >100 | 41 ± 18 | <10 | 45 ± 9 | 10 ± 5 |
| **14** | 12 ± 3 | 21 ± 7 | 22 ± 11 | 59 ± 13 | 19 ± 2 | 26 ± 5 | 28 ± 8 | 80 ± 10 | 38 ± 18 | 24 ± 7 | 36 ± 7 | 16 ± 5 |
| **15** | <10 | 10 ± 7 | 19 ± 7 | 39 ± 14 | <10 | <10 | <10 | 41 ± 18 | 12 ± 6 | <10 | 25 ± 10 | <10 |
| **16** | 11 ± 3 | <10 | 15 ± | <10 | <10 | >100 | 21 ± 6 | <10 | 21 ± 9 | <10 | nd | nd |
| **17** | <10 | <10 | <10 | 53 ± 5 | <10 | <10 | <10 | >100 | 22 ± 4 | <10 | 21 ± 10 | <10 |
| **18** | 13 ± 6 | 15 ± 2 | 22 ± 9 | 33 ± 6 | 22 ± 2 | 28 ± 4 | 14 ± 8 | 75 ± 9 | 17 ± 8 | 19 ± 5 | 34 ± 8 | 15 ± 5 |
| **19** | 13 ± 4 | 20 ± 5 | 36 ± 14 | 60 ± 11 | 19 ± 1 | 22 ± 2 | 24 ± 6 | 70 ± 5 | 26 ± 12 | 20 ± 6 | 40 ± 8 | 15 ± 3 |
| **20** | 15 ± 2 | 17 ± 3 | 20 ± 3 | 48 ± 9 | 10 ± 5 | 11 ± 4 | <10 | >100 | 32 ± 6 | <10 | 35 ± 8 | 14 ± 6 |
| **21** | 16 ± 4 | 14 ± 2 | 13 ± 6 | 26 ± 9 | 17 ± 7 | 12 ± 3 | 17 ± 5 | 49 ± 11 | 16 ± 7 | 15 ± 2 | 26 ± 4 | <10 |
| **22** | 15 ± 1 | 14 ± 8 | 16 ± 10 | 44 ± 8 | 20 ± 2 | 35 ± 5 | 23 ± 9 | 75 ± 8 | 23 ± 10 | 24 ± 4 | 31 ± 7 | 18 ± 2 |
| **23** | >100 | >100 | >100 | >100 | 77 ± 4 | 95 ± 4 | 81 ± 4 | >100 | >100 | >100 | >100 | 62 ± 4 |
| **24** | <10 | 15 ± 4 | 22 ± 6 | 54 ± 15 | 12 ± 4 | <10 | 11 ± 0 | 65 ± 20 | 20 ± 4 | <10 | 31 ± 10 | 13 ± 6 |
| **25** | 16 ± 3 | 10 ± 2 | 28 ± 2 | 66 ± 13 | <10 | 8 ± 3 | 10 ± 5 | >100 | 54 ± 19 | 18 ± 4 | 77 ± 3 | <10 |
| **26** | 70 ± 2 | 42 ± 5 | 79 ± 4 | 61 ± 1 | 49 ± 8 | 75 ± 1 | 66 ± 5 | 72 ± 3 | 42 ± 3 | 59 ± 2 | 60 ± 3 | 65 ± 4 |
| **27** | 76 ± 2 | 54 ± 5 | 65 ± 2 | 70 ± 3 | 48 ± 3 | 78 ± 1 | 57 ± 7 | 98 ± 4 | 38 ± 3 | 70 ± 1 | 61 ± 6 | 63 ± 1 |
| **28** | 16 ± 2 | 12 ± 9 | 27 ± 1 | 52 ± 17 | 12 ± 5 | <10 | 12 ± 1 | 59 ± 23 | 18 ± 3 | <10 | 36 ± 8 | <10 |
| **29** | 15 ± 1 | 14 ± 4 | 27 ± 5 | 77 ± 5 | 10 ± 4 | 14 ± 3 | 10 ± 2 | >100 | 38 ± 9 | <10 | 72 ± 9 | 21 ± 8 |

**Table S2.** Percentage of Cell Growth Inhibition in response to 25μM of Drug- Library B

|  | **HT29^a^** | **U87^b^** | **MCF-7^c^** | **A2780^d^** | **H460^e^** | **A431^f^** | **Du145^g^** | **BE2-C^h^** | **SJ-G2^b^** | **MIA^i^** | **SMA^j^** | **MCF10A^k^** |
| --- | --- | --- | --- | --- | --- | --- | --- | --- | --- | --- | --- | --- |
| **30** | 73±4 | 52±5 | >100 | >100 | 26±8 | 59±6 | 35±5 | >100 | >100 | 63±5 | >100 | >100 |
| **31** | 73±3 | 53±3 | 73±5 | 55±9 | 52±9 | 75±2 | 83±2 | 68±3 | 56±6 | 72±2 | 54±4 | 66±2 |
| **32** | 15±4 | 19±2 | 22±2 | 60±17 | 20±8 | 17±2 | 18±4 | 27±15 | 19±3 | 13±1 | 58±13 | 58±15 |
| **33** | 53±2 | 43±11 | >100 | 98±0.5 | 45±2 | 57±3 | 40±1 | 81±1 | 92±5 | 72±2 | 81±10 | 86±3 |
| **34** | 82±2 | 61±4 | >100 | 100±1 | 49±9 | 85±2 | 41±3 | >100 | 83±2 | 70±1 | 98±1 | 99±2 |
| **35** | 59±3 | 38±2 | 87±1 | 66±1 | 61±3 | 49±3 | 56±2 | 61±2 | 67±4 | 61±2 | 79±5 | 47±5 |
| **36** | >100 | 57±2 | >100 | 87±2 | 65±3 | 78±2 | 51±3 | >100 | 78±5 | 78±2 | 84±7 | 77±4 |
| **37** | >100 | 74±3 | 88±4 | 76±3 | 95±1 | >100 | >100 | >100 | >100 | 91±2 | 85±3 | >100 |

**Table S3.** Percentage of Cell Growth Inhibition in response to 25 μM of Drug- Library C

|  | **HT29^a^** | **U87^b^** | **MCF-7^c^** | **A2780^d^** | **H460^e^** | **A431^f^** | **Du145^g^** | **BE2-C^h^** | **SJ-G2^b^** | **MIA^i^** | **SMA^j^** | **MCF10A^k^** |
| --- | --- | --- | --- | --- | --- | --- | --- | --- | --- | --- | --- | --- |
| **38** | 59±4 | 67±5 | 57±3 | 63±4 | 51±2 | 81±0.3 | 56±2 | 28±12 | 50±2 | 67±3 | 73±1 | 69±3 |
| **39** | 76±2 | 75±1 | 69±1 | 78±2 | 59±3 | 76±3 | 75±1 | 73±3 | 59±1 | 69± 2 | 54±11 | 78±1 |
| **40** | 77±2 | 74±1 | 70±2 | 79±1 | 61±3 | 81±1 | 75±1 | 74±1 | 58±2 | 71±2 | 59±8 | 78±1 |
| **41** | 75±2 | 82±3 | 70±3 | 68±2 | 62±1 | 90±1 | 71±2 | 74±7 | 60±2 | 76±1 | 68±6 | 83±2 |
| **42** | 71±3 | 69±4 | 77±4 | 79±1 | 63±3 | 81±1 | 70±3 | 56±4 | 52±1 | 76±2 | 56±9 | 76±1 |
| **43** | 81±2 | 91±1 | 77±2 | 94±1 | 76±1 | 92±1 | 80±2 | 44±6 | 55±1 | 90±2 | 80±7 | 89±2 |

**Table S4**. Calculated physicochemical properties for selected bolinaquinone analogues: **1**, **12**, **27**, **30**, **34**, **37**, **41** and **43**.^a^

|  | **Mol Wt** | **HBA** | **HBD** | **MolLogP** | **PSA (Å^2^)** |
| --- | --- | --- | --- | --- | --- |
| **1 (bolinaquinone)** | 358.21 | 4 | 1 | 5.10 | 48.97 |
| **12** | 244.07 | 4 | 0 | 1.85 | 41.65 |
| **27** | 320.10 | 4 | 0 | 3.65 | 41.38 |
| **30** | 320.10 | 4 | 0 | 3.45 | 41.90 |
| **34** | 430.14 | 6 | 0 | 4.84 | 56.81 |
| **37** | 446.15 | 4 | 0 | 6.71 | 41.36 |
| **41** | 374.20 | 2 | 2 | 5.21 | 48.14 |
| **43** | 474.23 | 2 | 2 | 7.91 | 46.73 |

^a^ Properties calculated using Molsoft’s – Drug-likeness and molecular properties prediction (http://molsoft.com/mprop/)


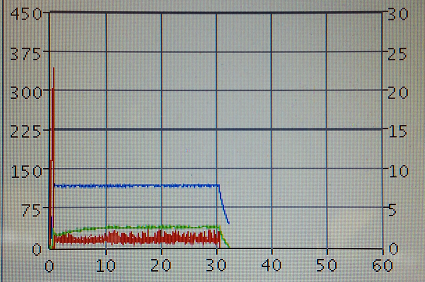


Figure S1. Representative graph of the temperature/power/pressure for the microwave-mediated Suzuki coupling. Blue: Temperature (°C); Green: Pressure (bar); Red: Power (watt). Reaction time: 20 min.

**Spectra for compounds synthesised.**

DMSO

H_2_O


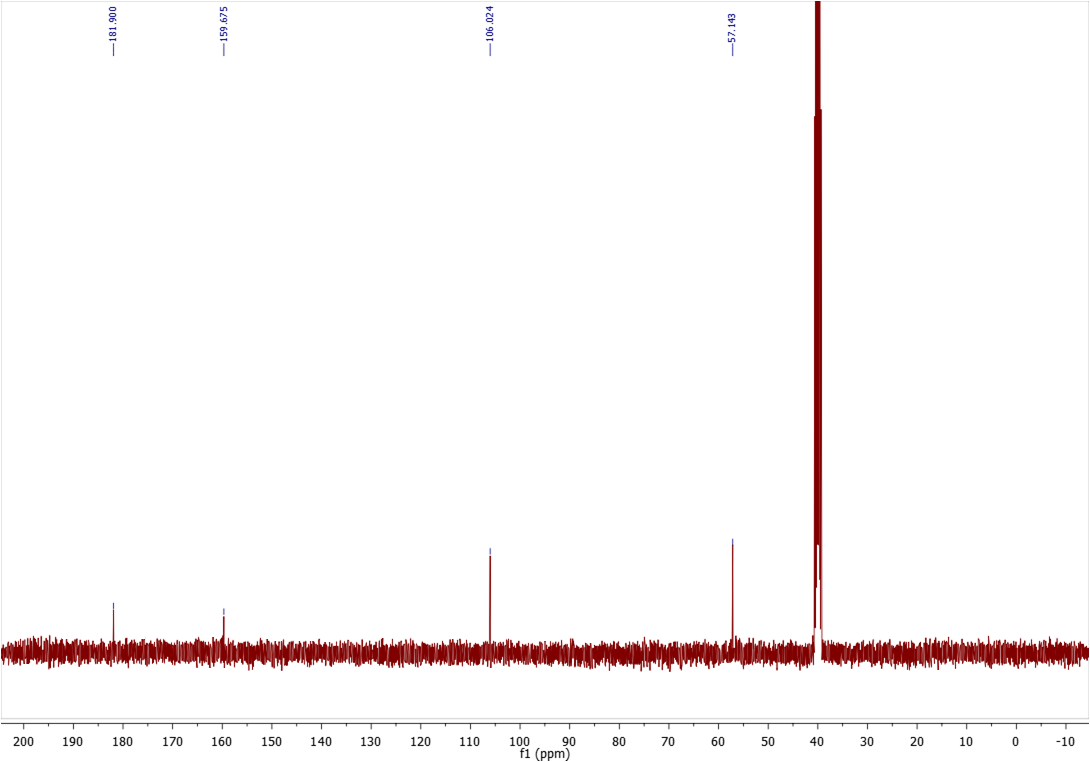


H2O

Acetone


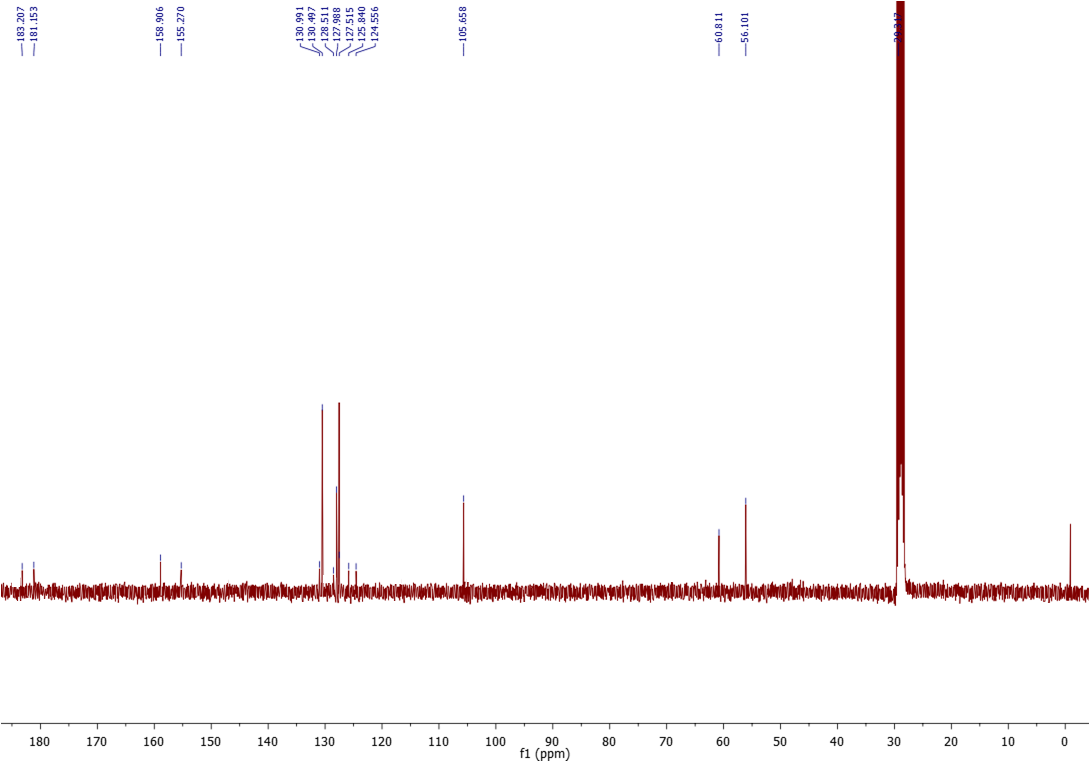

Supplement: Supporting Data [file rsos171189supp1.doc]
